# Cost-effectiveness of COVID-19 vaccination in Latin America and the Caribbean: an analysis in Argentina, Brazil, Chile, Colombia, Costa Rica, Mexico, and Peru

**DOI:** 10.1186/s12962-023-00430-2

**Published:** 2023-04-01

**Authors:** Augustovski Federico, Bardach Ariel, Santoro Adrián, Rodriguez-Cairoli Federico, López-Osornio Alejandro, Argento Fernando, Havela Maissa, Blumenfeld Alejandro, Ballivian Jamile, Solioz Germán, Capula Analía, López Analía, Cejas Cintia, Savedoff William, Palacios Alfredo, Rubinstein Adolfo, Pichon-Riviere Andrés

**Affiliations:** 1 Departamento de Evaluación de Tecnologías Sanitarias y Economía de la Salud/Health Technology Assessment and Health Economics Department/ Instituto de Efectividad Clínica y Sanitaria (IECS)/Institute for Clinical Effectiveness and Health Policy, Dr. Emilio Ravignani 2024 (C1014CPV), Buenos Aires, Argentina; 2 Centro de Implementación e Innovación en Políticas de Salud (CIIPS). Instituto de Efectividad Clínica y Sanitaria (IECS)/Institute for Clinical Effectiveness and Health Policy, Buenos Aires, Argentina; 3 Social Insight, Arrowsic, ME USA

## Abstract

**Objective:**

Our study analyzes the cost-effectiveness of the COVID-19 vaccination campaigns in Argentina, Brazil, Chile, Colombia, Costa Rica, Mexico, and Peru.

**Methods:**

Using a previously published SVEIR model, we analyzed the impact of a vaccination campaign (2021) from a national healthcare perspective. The primary outcomes were quality adjusted life years (QALYs) lost and total costs. Other outcomes included COVID-19 cases, hospitalizations, deaths, and life years. We applied a discount rate of 3% for health outcomes. We modeled a realistic vaccination campaign in each country (the realistic country-specific campaign). Additionally, we assessed a standard campaign (similar, “typical“ for all countries), and an optimized campaign (similar in all countries with higher but plausible population coverage). One-way deterministic sensitivity analyses were performed.

**Findings:**

Vaccination was health improving as well as cost-saving in almost all countries and scenarios. Our analysis shows that vaccination in this group of countries prevented 573,141 deaths (508,826 standard; 685,442 optimized) and gained 5.07 million QALYs (4.53 standard; 6.03 optimized). Despite the incremental costs of vaccination campaigns, they had a total net cost saving to the health system of US$16.29 billion (US$16.47 standard; US$18.58 optimized). The realistic (base case) vaccination campaign in Chile was the only scenario, which was not cost saving, but it was still highly cost-effective with an ICER of US$22 per QALY gained. Main findings were robust in the sensitivity analyses.

**Interpretation:**

The COVID-19 vaccination campaign in seven Latin American and Caribbean countries -that comprise nearly 80% of the region- was beneficial for population health and was also cost-saving or highly cost-effective.

**Supplementary Information:**

The online version contains supplementary material available at 10.1186/s12962-023-00430-2.

## Introduction

Since its emergence in December 2019 in Wuhan, China [1], the SARS-CoV-2 virus has spread rapidly. By March 2022, reported cases of SARS-Cov-2 exceeded 446 million worldwide, and reported deaths had exceeded six million people [2]. Latin America and the Caribbean (LAC) is one of the regions in the world with the highest number of deaths [3]. The lack of evidence-based guidelines to lead public policies has hindered the implementation of adequate control and mitigation measures both globally as in the Latin American region.

An essential component of evaluation and decision-making in relation to different health policies lies in assessing their efficiency (or cost-effectiveness). Vaccination for the recent SARS-CoV-2 pandemic has generally been implemented without formally assessing this dimension, given the global emergency it caused. More recently, several cost-effectiveness analyses of COVID-19 vaccination have been published [4–12], most of them focused on high-income countries and only one with a specific focus on a Latin American country [13]. Although there is much experience in developing and conducting economic evaluations in high-income countries, this is not the case for low- and middle-income countries. Paradoxically, those countries with more resources have already established this type of analysis for health decision-making, while in countries that need it most due to their scarcity of resources, its development and use is deficient [14]. So far it is unclear how appropriate it would be to carry out indirect estimates from the findings of economic evaluations conducted in high-income countries for its application in low- and middle-income settings. This is fundamentally due to, among other aspects, different social and macroeconomic contexts, different ways of coverage by third-party health payers, different absolute and relative costs of health care technologies and disease priorities [14].

Our team has worked on modeling the COVID epidemic in Latin America and the Caribbean (LAC) since the beginning of the pandemic with various projects and funding sources (Inter-American Development Bank -IDB-, World Health Organization -WHO-, and Argentine National Scientific and Technical Research Council -CONICET-). We initially developed a compartmental SEIR model (susceptible, exposed, infected, recovered) to assess the impact of the first wave and public health and social measures in a group of regional countries, then extended it to 26 LAC countries, and finally transformed it into an SVEIR (susceptible, vaccinated, exposed, infected, recovered) model to incorporate the effectiveness of vaccination. Although these models are computationally more complex than static models, they allow modeling key parameters of the dynamics of infectious diseases such as the transmissibility of the infection, the influence of natural immunity, the benefit of herd immunity, among other parameters [15]. More details of these projects can be found on the official webpage where the interactive model is available and in a peer-reviewed article recently published [16–18].

In the present study, our objective is to evaluate the cost-effectiveness of vaccination for Covid-19 in Argentina, Brazil, Chile, Colombia, Costa Rica, Mexico, and Peru.

## Methods

We used the CHEERS 2022 Checklist (Consolidated Health Economic Evaluation Reporting Standards) to guide this study report [19, 20]. A health economic analysis plan was undertaken and agreed with the sponsor (Inter-American Development Bank—IADB) at the start-up of the project and in the first interim report [21].

Although the original SVEIR model target population was the general population of Argentina, Brazil, Chile, Colombia, Costa Rica, Peru, and Mexico, this analysis only included the adult population (more than 18 years old). The study estimated the impact of vaccines on adults in each country, i.e., at the national level. The scenarios for different vaccination campaigns were constructed in accordance with the national health systems of each country, adapting the parameters to reflect local health resources and capacities and adopting a healthcare system perspective.

The base case analysis aimed to answer the following question: “how cost-effective were vaccination campaigns in each country analyzed when considering the most country-specific data available (type of vaccine applied, coverage and costs)?” Thus, for each country, it compared the implementation of a country-specific vaccination campaign to a policy of not vaccinating.

In addition to the base case analysis, we analyzed two additional scenarios to answer two further ancillary questions. First, to ease comparability, how cost-effective would it be if a “typical” vaccination campaign was applied in this set of Latin American and Caribbean countries? This simplistic “standard” scenario assessed the same “typical” vaccination campaign for all countries regarding the level of overall vaccine mix, efficacy, coverage, and vaccination costs, using weighted average data from the campaigns carried out in the region's countries. Second, we aimed to estimate what would have been the cost-effectiveness of an “optimized” campaign in each country. In this optimized scenario we modeled a hypothetical but plausible campaign-similar in all countries—in which the most effective vaccine was used, and the highest plausible uptake data from the region (Chile [22]).

In both the base case as well and in the additional scenarios, both arms of the comparison assumed similar public health and social measures with an intermediate level of stringency, including using face masks and physical distancing in closed environments.

All vaccination campaign scenarios were modeled for a 1-year time horizon (January 1—when vaccines became available in our region—to December 31, 2021); however, the health outcomes include long-term consequences measured by the quality-adjusted life expectancy loss in each strategy for the affected population.

No discount rate was applied to events and costs that occurred during the year of analysis. However, a discount rate of 3% was used (as recommended by the Bill & Melinda Gates Reference Case) for calculating health outcomes from subsequent quality adjusted years of life [23]. Long-term costs and non-fatal consequences of COVID-19 are not included because their frequency and impact in the medium and long term are still uncertain, which is probably why no economic evaluation published to date includes them. The primary health outcomes were the quality-adjusted life years (QALYs) lost for each strategy (and to characterize the gains with vaccination). Additionally, deaths, years of life lost, and COVID-19 cases (critical, severe, symptomatic) averted were reported.

Throughout the project we had an international advisory board with different regional stakeholders to guide this exercise to the best regional decision-making process (see its composition in the acknowledgements section and more in the Additional file 1).

### Epidemiological, quality of life, vaccine coverage and efficacy parameters. Resource use and costs

A literature search was performed in the Medline and Lilacs databases (see Additional file 1) for parameters related to COVID-19 and health service use (days of hospitalization, percentage of patients requiring hospitalization in general wards and intensive care units, mortality rates, among others). We also searched for information on the efficacy and effectiveness of the COVID-19 vaccines (in clinical trials and real-world studies). In addition, official websites of ministries of health were searched for country-specific information on vaccination campaigns, prioritizing the soundest methodological approaches for the different parameters. In the case of official and public websites, we decided to use what, in our opinion, were the more reliable and rigorous sources of information on key parameters. Table 1 shows epidemiological and cost parameters, including assumptions and sources.

**Table 1:** Study assumptions and design, epidemiological, utility and cost parameters *Sources*: [1] Oran DP, Topol EJ. Prevalence of Asymptomatic SARS-CoV-2 Infection: A Narrative Review. Ann Intern Med. 2020;173(5):362–367. 10.7326/M20-3012. [2] Lapidus N, Paireau J, Levy-Bruhl D, de Lamballerie X, Severi G, Touvier M, Zins M, Cauchemez S, Carrat F; SAPRIS-SERO study group. Do not neglect SARS-CoV-2 hospitalization and fatality risks in the middle-aged adult population. Infect Dis Now. 2021 Jun;51(4):380–382. 10.1016/j.idnow.2020.12.007. Epub 2021 Jan 18. PMID: 33,521,775; PMCID: PMC7836556. [3] Almeshari M, Alobaidi N, Al Asmri M, et alP61 Mechanical ventilation utilization in COVID-19: a systematic review and meta-analysisThorax 2021;76:A121. [4] NF Brazeau, R Verity, S Jenks et al. COVID-19 Infection Fatality Ratio: Estimates from Seroprevalence. Imperial College London (29–10-2020), https://doi.org/10.25561/83545. [5] Peak CM, Kahn R, Grad YH, Childs LM, Li R, Lipsitch M, Buckee CO. Individual quarantine versus active monitoring of contacts for the mitigation of COVID-19: a modelling study. Lancet Infect Dis. 2020 Sep;20(9):1025–1033. 10.1016/S1473-3099(20)30361-3. Epub 2020 May 20. PMID: 32445710; PMCID: PMC7239635. [6] CDC. COVID-19 Pandemic Planning Scenarios. Updated Mar. 19, 2021. Available at: https://www.cdc.gov/coronavirus/2019-ncov/hcp/planning-scenarios.html. Accessed on May 2022. [7] Estenssoro E, Loudet CI, Ríos FG, Kanoore Edul VS, et al. SATI-COVID-19 Study Group. Clinical characteristics and outcomes of invasively ventilated patients with COVID-19 in Argentina (SATICOVID): a prospective, multicenter cohort study. Lancet Respir Med. 2021 Sep;9(9):989–998. 10.1016/S2213-2600(21)00229-0. Epub 2021 July 2. PMID: 34224674; PMCID: PMC8253540

General Inputs and study-model attributes and assumptions	Base case value (range if it was incorporated in the sensitivity analysis)	Source
Time horizon	1 year: January to December 31 2021	Time horizon: assumption based on previous economic evaluations [5, 6, 8, 10]
Cycle length	1 day	N/A
Perspective	National Healthcare System	N/A
Annual discount rate (only for life expectancy and QALYs)	3%	Bill & Melinda Gates Reference Case [23]
Primary health benefit outcome	QALY loss	N/A
Secondary health benefit outcomes	Deaths, Years of life lost, COVID-19 cases, COVID-19 Hospitalizations (general ward and ICU)	N/A
*Average population distribution at baseline*		
Susceptible (%)	94.20%	SVEIR model data on January first, 2021
Exposure (%)	0.10%	SVEIR model data on January first, 2021
Infected (%)	0.10%	SVEIR model data on January first, 2021
Recovered (%)	5.60%	SVEIR model data on January first, 2021
Average proportion of asymptomatic subjects among not hospitalized infected	0.45	Oran et al. [1]
Average COVID-19 Hospitalization rate on general ward per 10,000 infected subjects	3.5*	Lapidus et al. [2]
Average COVID-19 Hospitalization rate on ICU per 10,000 infected subjects	1.8*	Lapidus et al. [2]
Average proportion of ICU patients with invasive mechanical ventilation	0.71	Almeshari et al. [3]
Infectious fatality rate-IFR—% by age group	18–29: 0.03%; 30–39: 0.07%; 40–49: 0.19%; 50–59: 0.46%; 60–69: 1.12%; 70–79: 2.68%; > 80: 7.97%	Brazeau et al. [4]
*Immunity protection length*		
Natural immunity protection—time in days	180	Assumption
Two doses vaccine immunity protection—time in days	360 (270–360)	Assumption (**)
*Length of symptoms duration or hospitalization (in days)*		
Symptomatic case w/o hospitalization	4.8	Peak et al. [5]
Hospitalization in general Ward	5	CDC report [6]
Hospitalization in ICU	17	Estenssoro et al. [7]
Population Health Utility (by country and age)	See Additional file 1	See Additional file 1
*Proportional utility decrements from age adjusted population values*		
Symptomatic case w/o hospitalization	0.19 loss	Kohli et al. [5, 6, 8, 10]
Hospitalization	0.30 loss	Kohli et al. [5, 6, 8, 10]
UCI w/o mechanical ventilation	0.50 loss	Kohli et al. [5, 6, 8, 10]
UCI with mechanical ventilation	0.60 loss	Kohli et al. [5, 6, 8, 10]
*Average resource cost per event/per day—USD*		
Cost per each COVID-19 case diagnosed (***)	ARG: $100.20; BR: $95.29; CL: $127.87; COL: $156.99; CRI: $122.18; MEX: $70.97; PE: $185.34	Own estimation (see “Methods” section)
Symptomatic case w/o hospitalization event cost (USD)	ARG: $116.5; BR: $105.2; CL: $166.8; COL: $179.6; CRI: $147.1; MEX: $150.7; PE: $195.6	Own estimation (see “Methods” section)
Hospitalization on general ward cost per day (USD)	ARG: $130.1; BR: $26.0; CL: $159.1; COL: $213.2; CRI: $127.0; MEX: $444.5; PE: $224.6	Own estimation (see “Methods” section)
Hospitalization on ICU w/o invasive mechanical ventilation cost per day (USD)	ARG: $239.2; BR: $236.4; CL: $191.3; COL: $397.8; CRI: $356.1; MEX: $2116.6; PE: $366.7	Own estimation (see “Methods” section)
Hospitalization on ICU with invasive mechanical ventilation cost per day (USD)	ARG: $263.0; BR: $251.2; CL: $200.0; COL: $408.9; CRI: $377.1; MEX: $2368.2; PE: $384.2	Own estimation (see “Methods” section)

Costs are expressed in American dollars for November 2021

(*) For specific age disaggregated data see Additional file 1. (**) We assumed that vaccine efficacy does not wane during the time horizon of the analysis

We based this assumption on other economic evaluations in the field. (***) We assume that for each covid case diagnosed by nasopharyngeal swab, 5 nasopharyngeal swabs were carried out, 4 of which were negative

#### COVID-19 disease-related parameters

The main parameters in the model related to COVID-19 were population rates of different disease states (cases, symptomatic disease without the requirement of hospitalization, hospitalization in the general ward, hospitalization in the intensive care unit (ICU), and death) (see Additional file 1).

#### COVID-19 vaccine-related parameters

We obtained data on the effectiveness or efficacy of all COVID-19 vaccines that have been used in the seven countries (see Additional file 1: Table S1). We estimated the weighted average effectiveness value of the vaccination campaign per country and the number of doses given. We calculated this using the mix of the different vaccines that each country had used (see Additional file 1). We used this data to populate the country-specific realistic base case analysis. For the standard vaccination campaign scenario, we used the average vaccine effectiveness of the seven countries. Finally, we selected the higher vaccine effectiveness values available per outcome (and per total number of doses) to populate the optimized scenario.

#### Other epidemiological and transmission dynamic parameters

Regarding disease transmission dynamics, the model establishes the number of infections through different transmission probability values according to age groups using information for contact matrices and effective contact matrices (see Additional file 1 for more details). The reproduction value (R0) based on contact matrices represents the number of contacts arising from the interaction between the different age groups in different settings: home, work, schools, and community. This defines how effective (in terms of contagiousness) these contacts are. More detailed information on the model is available in a recently published manuscript [18]. Finally, we assumed that both the intervention strategy (all the scenarios) and the non-vaccination strategy implemented public health and social measures with an intermediate level of stringency (use of face masks and physical distancing in closed environments), based on data published by Davis et al. [24].

#### Vaccine coverage

We used reported country-specific data on vaccine coverage for the base case realistic analysis [25]. For the standard scenario, we calculated a simple average from the coverage values of each country for both one and two doses. For the optimized coverage scenario with two doses, the highest coverage value reported in the region was chosen as a benchmark. In order to derive the value of coverage of the first dose, we applied the same ratio (of second to two dose coverage) observed in the standard scenario.

#### Utility values

To estimate QALYs, we used age and country-specific life tables as well as population utility values (see Additional file 1) [26]. COVID-specific impact was thus captured as the short term quality of life loss during the 1 year time horizon (in case of the non-fatal events) and the QALYs lost in case of fatal events. These QALYs were calculated for each death based on the age at which death occurred and the quality of life of the general population by age.

The impact of adverse events of vaccination was not incorporated (these are usually minor and were not included in most existing economic evaluations). To incorporate baseline utilities by age group and country we prioritized utility data obtained through the “time-trade off” (TTO) methodology [26]. If the case data based on TTO were not available and the "visual analog scale" methodology was available, the formula for converting utility values from one scale to another reported by Stiggelbout et al. was used [27].

Additionally, specific utility/disutility values for each health state were incorporated. Among all the economic evaluations for Covid-19 vaccines identified, four of them report quality-adjusted life years lost [5, 6, 8, 10]. After reviewing these studies we found it more appropriate to use the proportional utility decrements from age-adjusted population values reported in the Kohli 2021 study [5, 6, 8, 10] (see Additional file 1 for detailed information).

#### Health system costs

The health system costs considered three major components: (i) the costs of vaccination, (ii) the costs of health events associated with COVID-19, and (iii) the costs related to testing. The methodological approach to the cost of each of these components, as well as the data sources, are briefly described below (for further details see Additional file 1). All costs were expressed in US dollars for November 2021.

##### Vaccination costs

For each of the study countries, we estimated a weighted average cost per vaccine applied. We followed an approach based on three stages. First, we identified the acquisition cost of each of the vaccines administered in each country based on official information and technical documents for each country. Second, we estimated the costs related to logistics, storage, and distribution of the vaccines in each country based on official information and technical documents for some countries, and indirect estimations for other countries. These costs were added to the vaccine acquisition costs to get a proxy of the cost by vaccine applied in each country. Third, we constructed analytic weights for each vaccine based on the number of doses applied in each country. The weighted average cost per vaccine applied in each country was equal to the average cost per vaccine applied weighted by the corresponding analytical weight. For further details see Additional file 1.

##### Costs of health events associated with COVID-19

We considered the following health event costs associated with a case of COVID-19: symptomatic ambulatory event, symptomatic hospitalized event, hospitalization in intensive care without mechanical ventilation (MV) event, and hospitalization in intensive care with MV event. The unit costs necessary to estimate these health event costs (mainly the cost per day in a general ward bed, in intensive care with MV, and in intensive care without MV) were obtained from official information on unit costs (i.e., official nomenclatures and tariffs) in the public and/or social security sector in each country. In countries where some of the unit costs could not be obtained from official information, we conducted a literature review to identify the unit cost information required by our model. For further details see the Additional file 1.

##### Testing costs

We collected official information on testing kit purchases in each country. In those countries where access to such information was not available, we analyzed publications in specialized journal sites and conducted indirect estimates for testing costs based on information from countries with data. The costs of testing were incorporated into the cost of the symptomatic patient state by incorporating an average rate of testing per symptomatic case. For further information see the Additional file 1.

### SVEIR model

The model used for this study was developed by our team at the Institute for Clinical and Health Effectiveness (IECS) in Argentina with the support of the Inter-American Development Bank (IDB) and entitled "Integrated Model of Preparedness and Response of Health Systems in Latin America and the Caribbean to estimate the impact of COVID-19 expansion" [18]. This user-friendly, open source, transparent and interactive model was developed to facilitate the decisions of policy makers by allowing the user to modify its parameters according to the specific pandemic trajectory, policy context and vaccination strategy in each country.

**Fig. 1:**
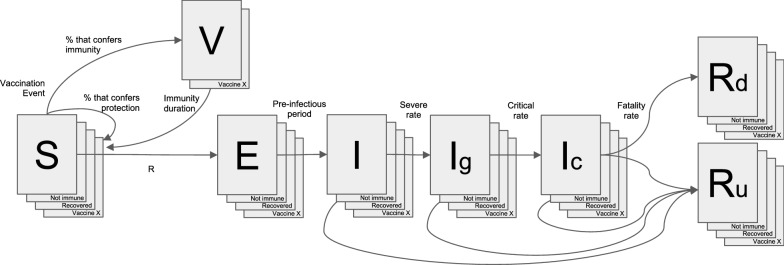
SVEIR model scheme. S: susceptible; V: vaccinated; E: exposed; I: infectious (Ig: Infectious in general ward; Ic: infectious in critical care), Rd: death; Ru: recovered

More details about the interactive open source model including its calibration can be found in the publication and on the model's website [28]. The SVEIR model presented here added a new transition state to the SEIR model, the vaccinated population compartment (V), as well as incorporating compartments by age groups. It also more accurately gauges the trajectory of the epidemic by incorporating public health and social measures, as well as epidemiological and clinical data from each country. To model the impact of vaccination strategies, a representation of different states of immunity, both related to vaccination and natural immunity, were added. The model scheme is presented in Fig. 1. For the visualization of the model, an interactive application programmed in R with a visual user interface was developed in Shiny.js. This application provides access to epidemic projections for 26 countries (including the countries of this study). It uses daily epidemiological information on deaths reported by each country. Therefore, the number of new cases per day is inferred through the number of deaths reported, divided by the estimated Infectious Fatality Rate (IFR).

After running the model for each country and each comparator in the base case and the different alternative vaccination scenarios, the health outcomes and costs of each comparator were reported. The analysis also reports the differences in health and costs, along with the incremental cost-effectiveness ratio (ICER) if relevant (i.e., vaccination was more effective and more costly).

### Incorporating uncertainty

We assessed uncertainty in our results using several complementary approaches. First, we incorporated two additional scenarios besides the realistic base case analysis: a standard and an optimized vaccination campaign scenario, which tackle different research questions described above. Second, we conducted selected one-way sensitivity analyses for parameters that were found to be most influential in the literature, such as vaccine dose cost and effectiveness parameters, using the best available evidence to define the uncertainty intervals. Third, the model is not only in the public domain and openly available; it also provides users with the opportunity to select and customize their own parameter values and thereby adapt the model to address the most relevant questions in their local context, and to perform multiple scenario sensitivity analyses in an interactive user-defined way [28].

We carried out the one-way deterministic sensitivity analysis per each country and scenario. Uncertainty in the following parameters was considered in this sensitivity analysis: effectiveness or efficacy of vaccines, vaccination coverage, vaccine costs, costs of health events, disease transmission and length of protection time for vaccine immunity.

For the data on the effectiveness or efficacy of the vaccines, an assumption was made based on the 95% confidence intervals reported in those studies from which the central estimates were obtained. Specifically, we took into consideration the reported variability (95% confidence intervals) for those vaccines most used in the region (AstraZeneca, Pfizer, and Sinovac), which represented 83% of the present analysis. From the studies by Voysey et al. (AstraZeneca) [29], Jara et al. (Sinovac) [30], and Hass et al. (Pfizer) [31], it was found that the relative difference between the extremes of the 95% confidence interval in relation to the central estimate for the outcome variable of infection (2nd dose) was 12%, 1%, and 0.3%, respectively. Consequently, and to be conservative, we chose to apply the higher variability reported (12% in relative terms by Voysey et al. [29], AstraZeneca) for all the effectiveness/efficacy vaccine-related outcomes included in the model, for one and two doses.

For the remaining parameters, and given the lack of information to estimate uncertainty values, we decided to choose a range of ± 25% in relation to the central value, as suggested by the economic evaluation literature [32, 33]*.* The evaluation of distributional effects or costs was not incorporated.

## Results

We summarize the estimates and sources of the main model parameters in Table 1. According to the SVEIR model, 94% of the population was susceptible, and 5.6% had recovered from infection at the beginning of the analysis. Table 2 shows vaccine-related inputs used in this analysis (coverage, effectiveness, and vaccine cost values data) and the respective sources for each of the seven countries analyzed (for the base case analysis as well as for standard and optimized scenarios).

**Table 2:** Efficacy and costs of vaccination programs (weighted average)

	Base case (realistic vaccination campaign)							Standard vaccination scenario (LAC)	Optimized vaccination scenario (LAC)
	Argentina	Brazil	Chile	Colombia	Mexico	Peru	Costa Rica		
*Vaccine coverage % (range for DSA)*									
1 dose	80.8 (60.6–100)	76.8 (57.6–96)	87.85 (65.89–100)	73.43 (55.07–91.79)	59.4 (44.55–74.25)	67.58 (50.69–84.48)	75.64 (56.73–94.55)	74.5 (55.88–93.13)	100 (75–100)
2 doses	65.79 (49.34–82.24)	63.24 (47.43–79.05)	84.14 (63.11–100)	50.03 (37.52–62.54)	48.27 (36.2–60.34)	55.69 (41.77–69.61)	62.56 (46.92–78.2)	61.4 (46.05–76.75)	87.85 (65.89–100)
*Vaccine effectiveness % (95% CI) 1 dose (range for DSA)*									
Symptomatic cases w/o hospitalization	74.46 (65.52–83.39)	73.48 (64.66–82.30)	64.48 (64.05–82.30)	73.15 (64.37–81.93)	75.91 (66.80–85.02)	81.05 (71.33–90.78)	75.74 (66.65–84.83)	71.14 (63–80)	93.00 (81.84–100)
Hospitalizations	87.01 (76.57–97.45)	72.78 (64.05–81.52)	47.42 (42.00–53.00)	64.54 (56.79–72.28)	76.19 (67.04–85.33)	76.63 (67.44–85.83)	77.87 (68.53–87.22)	65.92 (58–74)	100 (88–100)
UCI	97.30 (85.63–100)	66.43 (58.46–74.40)	51.51 (45.00–58.00)	62.82 (55.28–70.36)	77.30 (68.02–86.58)	77.30 (68.02–86.58)	74.56 (65.61–83.51)	62.05 (55–70)	100 (88–100)
Death	83.24 (73.26–93.23)	65.76 (57.86–73.65)	51.14 (45.00–57.00)	63.26 (55.67–70.85)	71.70 (63.10–80.30)	71.70 (63.10–80.30)	68.81 (60.55–77.06)	60.65 (53–68)	100 (88–100)
*Vaccine effectiveness % (95% CI) 2 doses (range for DSA)*									
Symptomatic cases w/o hospitalization	79.94 (70.34–89.53)	77.57 (68.26–100)	72.1 (63.45–80.75)	80.78 (71.09–90.48)	88.09 (77.52–98.66)	92.21 (81.15–100)	78.02 (68.66–87.39)	76.54 (67–86)	98.50 (86.68–100)
Hospitalizations	96.45 (84.88–100)	95.45 (84.00–100)	90.00 (79.20–100)	93.64 (82.40–100)	96.09 (84.56–100)	97.40 (85.71–100)	96.57 (84.98–100)	93.96 (83–100)	100 (88–100)
UCI	99.31 (87.40–100)	96.82 (85.20–100)	92.23 (81.17–100)	94.78 (83.40–100)	98.34 (86.54–100)	97.88 (86.13–100)	97.91 (86.16–100)	95.68 (84–100)	100 (88–100)
Death	99.35 (87.43–100)	95.63 (84.15–80.75)	89.08 (78.39–99.77)	93.10 (81.93–100)	97.93 (86.18–100)	97.22 (85.55–100)	97.22 (85.55–100)	91.70 (81–100)	100 (88–100)
Vaccine cost per dose—USD$ (range for DSA)	8.62 (6.47–10.78)	8.28 (6.21–10.35)	11.13 (8.35–13.91)	13.81 (10.36–17.26)	6.09 (4.57–7.61)	16.28 (12.21–20.35)	10.74 (8.06–13.43)	8.58 (6.44–10.73)	8.58 (6.44–10.73)
Total costs per each vaccine dose applied (*)—USD$ (range for DSA)	8.98 (6.74–11.23)	8.54 (6.41–10.68)	11.46 (8.6–14.33)	14.07 (10.55–17.59)	6.36 (4.77–7.95)	16.61 (12.46–20.76)	10.95 (8.21–13.69)	8.86 (6.65–11.08)	8.86 (6.65–11.08)

Base case (realistic vaccination campaign) and Standard and Optimized scenarios

Most of these values were obtained after calculating weighted averages or simple averages. Please see “Methods” section and Additional file 1 for methodological approach, sources, and disaggregated data

(*) Total costs per vaccine finally applied include the cost of vaccine per unit, logistics costs, storage and distribution costs which were already described in the “Methods” section

DSA: deterministic sensitivity analysis

Costs are expressed in US dollars for November 2021

In addition, Table 3 summarizes the results of the economic evaluation for the base case analysis (realistic “country-specific” campaign) as well as for the standard and optimized scenarios. Vaccination was health improving as well as cost-saving in almost all countries and scenarios. As expected, in all countries and scenarios, vaccination was health-improving in terms of QALY gains, deaths avoided, and other health outcomes avoided (see more detailed results by country, including undiscounted figures in Additional file 1). For the base-case analysis discounted QALYs gained by vaccination ranged from 49,625 in Costa Rica to 1,518,053 in Mexico. Except in Chile, vaccination was cost saving, ranging from US$266,754,782 in Argentina to US$9,689,633,010 in Mexico. For Chile, although there was a gain of QALYs (144,257), the net costs of the campaign were slightly higher than in the no vaccination scenario (US$3,167,869), with an incremental cost-effectiveness ratio of US$22 per QALY gained.

**Table 3:** Main cost-effectiveness results in the seven countries

Strategy	Outcome/country	Argentina	Brazil	Chile	Colombia	Costa Rica	México	Perú
No vaccination	Costs	$ 2,489,103,233	$ 14,899,414,477	$ 1,426,693,488	$ 5,213,429,639	$ 1,000,387,362	$ 24,460,665,837	$ 3,290,563,102
	QALYs lost*	1,678,317	3,650,818	423,399	1,878,855	130,323	4,304,472	2,121,919
	Deaths	160,172	330,091	38,672	157,566	10,442	385,521	183,735
Base case (realistic vaccination campaign**)	Costs saved	$ 266,754,782	$ 4,600,027,570	($ 3,167,869)	$ 942,846,561	$ 374,025,049	$ 9,689,633,010	$ 415,853,089
	QALYs gained*	737,438	1,282,144	144,257	632,457	49,625	1,518,053	707,797
	Deaths avoided	85,414	152,547	17,877	67,996	4,955	165,561	78,791
	Cost-effectiveness	Cost-saving	Cost-saving	ICER: $22 per QALY gained	Cost-saving	Cost-saving	Cost-saving	Cost-saving
Standard vaccination campaign***	Costs saved	$ 244,482,745	$ 4,650,194,020	$ 80,149,172	$ 1,220,452,465	$ 319,049,088	$ 9,291,807,794	$ 660,129,685
	QALYs gained*	579,592	1,101,496	108,161	541,621	41,203	1,519,802	639,047
	Deaths avoided	67,159	129,451	13,293	57,972	4,099	165,805	71,047
	Cost-effectiveness	Cost-saving	Cost-saving	Cost-saving	Cost-saving	Cost-saving	Cost-saving	Cost-saving
Optimized vaccination campaign****	Costs Saved	$ 329,514,258	$ 4,488,290,446	$ 102,289,321	$ 1,358,560,340	$ 440,842,304	$ 11,123,858,434	$ 739,338,504
	QALYs gained*	810,293	1,419,377	150,731	742,587	56,440	1,971,977	878,760
	Deaths avoided	93,945	170,268	18,624	80,150	5,651	218,125	98,679
	Cost-effectiveness	Cost-saving	Cost-saving	Cost-saving	Cost-saving	Cost-saving	Cost-saving	Cost-saving

Base case (realistic vaccination campaign), standard and optimized scenarios

*3% discount rate; **realistic vaccination campaign (weighted efficacy and costs by country-specific vaccine use, coverage, and costs); ***standard vaccination campaign: same vaccination campaign in all countries (weighted efficacy and costs); ****optimized but realistic campaign (see paper text for more details)

Costs are expressed in American dollars for November 2021

In the standard vaccination scenario, vaccination was cost-saving in all countries: discounted QALYs gained ranged from 41,203 in Costa Rica to 1,519,802 in México; and vaccination net cost savings ranged from US$80,149,172 in Chile to US$9,291,807,794 in Mexico. Finally, in the optimized vaccination scenario, vaccination was also universally cost saving: discounted QALYs gained ranged from 56,440 in Costa Rica to 1,971,977 in México; and vaccination net cost savings ranged from US$102,289,321 in Chile to US$11,123,858,434 in Mexico.

In the Additional file 1 we report disaggregated results for each of the countries, including years of life lost, total cases and their costs, symptomatic cases and their costs, general ward/intensive care unit hospitalizations and their costs, and costs of vaccination campaigns. Undiscounted and discounted results are presented as well as discounted results where applicable.

### Uncertainty analysis

To illustrate the one-way deterministic sensitivity analysis, Table 4 reports the results for each vaccination scenario in the case of Brazil. Although some parameters were more significant than others, all the vaccination scenarios were cost-saving when considering the uncertainty in the selected parameters. Health benefits were more sensitive to uncertainty in the probability of disease transmission and the percentage of vaccination coverage implemented, while cost differences were more sensitive to uncertainty in the probability of disease transmission and health events costs. Sensitivity analysis results for the other countries showed a similar pattern (see Additional file 1). In Chile’s case, we also found similar results than in the base case under the realistic campaign scenario in which ICER was not cost saving. However, in the sensitivity analysis, the ICERs were never higher than US$540 per QALY gained.

**Table 4:** Deterministic sensitivity analysis (example of results for Brazil)

	No vaccination	Base case (realistic vaccination campaign**)		Standard vaccination campaign***		Optimized vaccination campaign****	
		Outcomes	ICER	Outcomes	ICER	Outcomes	ICER
*Main results (QALYs lost and total costs)*							
Vaccine efficacy (range ∓ 12%)							
QALYs lost*	3,650,818 to 3,650,818	2,371,905 to 2,366,092	Cost-saving	2,554,111 to 2,545,448	Cost-saving	2,231,568 to 2,231,313	Cost-saving
Total costs	$14,899,414,477 to $14,899,414,477	$10,328,733,920 to $10,271,273,293		$10,292,861,063 to $10,209,303,909		$10,413,997,276 to $10,407,423,859	
Vaccination cost per dose, including total vaccination costs (range ∓ 25%)							
QALYs lost*	3,650,818 to 3,650,818	2,368,674 to 2,368,674	Cost-saving	2,549,322 to 2,549,322	Cost-saving	2,231,441 to 2,231,441	Cost-saving
Total costs	$14,899,414,477 to $14,899,414,477	$9,737,176,309 to $10,860,768,550		$9,793,338,127 to $10,704,273,833		$97,27,507,775 to $11,093,911,333	
Vaccination coverage (range ∓ 25%)							
QALYs lost*	3,650,818 to 3,650,818	2,573,443 to 2,182,900	Cost-saving	2,726,390 to 2,389,756	Cost-saving	2,452,015 to 2,034,425	Cost-saving
Total costs	$14,899,414,477 to $14,899,414,477	$10,152,618,903 to $10,549,018,547		$10,318,767,762 to $10,382,247,295		$10,114,708,594 to $10,757,417,545	
Health event costs (range ∓ 25%)							
QALYs lost*	3,650,818 to 3,650,818	2,368,674 to 2,368,674	Cost-saving	2,549,322 to 2,549,322	Cost-saving	2,231,441 to 2,231,441	Cost-saving
Total costs	$11,174,560,858 to $18,624,268,097	$8,286,025,443 to $12,311,919,417		$8,142,072,338 to $12,355,539,622		$8,491,233,945 to $12,330,185,164	
Disease transmission (range ∓ 25%)							
QALYs lost*	2,312,737 to 4,856,134	1,400,574 to 3,093,737	Cost-saving	1,542,160 to 3,285,146	Cost-saving	1,290,504 to 2,951,926	Cost-saving
Total costs	$7,667,170,115 to $21,085,912,835	$7,287,515,194 to $12,417,277,625		$7,163,837,041 to $13,021,191,154		$7,419,212,868 to $12,377,885,228	
Vaccine immunity duration (range 270–365 days)							
QALYs lost*	3,650,818 to 3,650,818	2,386,808 to 2,357,638	Cost-saving	2,566,343 to 2,539,071	Cost-saving	2,249,817 to 2,220,215	Cost-saving
Total costs	$14,899,414,477 to $14,899,414,477	$10,336,865,926 to $10,277,311,931		$10,307,730,018 to $10,216,440,444		$10,442,698,112 to $10,391,333,074	

No vaccination; Base case (realistic vaccination campaign), Standard and Optimized campaigns. For the other countries' results see Additional file 1

*3% discount rate; **real-life vaccination campaign (weighted efficacy and costs by country-specific vaccine use, coverage, and costs); ***standard vaccination campaign: same vaccination campaign in all countries (weighted efficacy and costs); ****optimized but realistic campaign (see paper text for more details)

Costs are expressed in American dollars for November 2021

## Discussion

To our knowledge, this is the first comprehensive analysis conducted in LAC that assessed the cost-effectiveness of COVID-19 vaccination campaigns in Argentina, Brazil, Chile, Colombia, Costa Rica, Mexico, and Peru. In all these countries, that comprise roughly 80% of the region population [34], vaccination was able to prevent a significant amount of COVID-related disease burden (regarding the number of cases, hospitalizations, critical cases, deaths, life years gained, and QALYs). In all countries vaccination was cost-saving or highly cost-effective, and the increased costs of vaccination campaigns were offset by the larger cost-savings from not having to care for people with cases of COVID, or the additional costs were small in relation to health gained and thus highly cost-effective (Chile). Thus, at current vaccine acquisition costs, they were clearly a good "health investment" (best buy).

One strength of our study is that it assessed three plausible scenarios regarding vaccination: the realistic base-case scenario that uses the most likely country-specific parameters; the standard scenario that facilitates cross-country comparisons; and the optimized scenario that assumes higher but attainable coverage in our region. It is estimated that vaccination during 2021 in these seven countries prevented 573,141 deaths (508,826 standard campaign; 685,442 optimized campaign); and gained 5.07 million QALYs (4.53 standard campaign; 6.03 optimized campaign). In terms of costs to the health systems, despite the incremental costs of vaccination campaigns, the model estimated a net cost savings of US$16.29 billion (US$16.47 standard, US$18.58 optimized). Likewise, the health benefits and costs saved would have been higher if the vaccination campaigns had been implemented in a more optimized way, as shown by our optimized vaccination scenario. Finally, the sensitivity analysis showed that the conclusions of the main analyses were robust, even considering the uncertainty of the key inputs of the model. The results were most sensitive to the estimates chosen for the probability of disease transmission and the percentage of vaccination coverage, though neither of these variables changed the main results and conclusions.

An additional strength of our work is that model parameters and specifications were selected using evidence synthesis methods and adapting as much as possible the values to local country settings in this heterogeneous group of countries. Thus, we believe our study results reflect the real spectrum of cost-effectiveness of COVID vaccinations in Latin America and the Caribbean region. Also, our study has a variety of outcomes incorporated and reported, beyond deaths, QALYs and costs. It disaggregates results for different health outcomes, including the number of total cases, symptomatic cases, cases hospitalized in general ward, and critical cases in intensive care units. Another important strength of our economic evaluation is that it is based on an epidemiologic open source and open access SVEIR model [28] that was intended for health authorities and decision makers, and targeted to visualize and project the effects of different policies at the country level in 26 Latin American and Caribbean Countries [18]. The mathematical methodology behind the SVEIR model offers to decision makers the possibility to make more accurate predictions about the impact of infectious diseases and consequently the effectiveness and cost-effectiveness of the vaccination campaign in that context [15].

Our work is in line with most economic evaluations of COVID-19 vaccination performed to date, either in peer-reviewed articles [7, 8, 11–13, 32, 33, 35, 35, 36], or pre-print [4]. In all of them, vaccination is shown to be either cost-effective-using commonly-used cost-effectiveness thresholds- or directly cost-saving. Usually, health technologies are initially available at a higher cost than in later periods [36]. If that is the case—and if the magnitude of benefits remains stable-cost-effectiveness of vaccines could probably improve over time.

Also, our findings concur with the few studies we identified in the region. Taborda et al. [35] reported results of a budget impact analysis of Covid-19 vaccination in six Latin American countries. They state that the vaccination campaign during 2021 was cost-saving in all the countries analyzed. In addition, Fernandes et al. [13] published a cost-utility analysis of three Covid-19 vaccines in Brazil. They reported cost-saving results for two of the three most common vaccines in that country (Oxford and Janssen vaccine). The third vaccine, CoronaVac, was not cost-saving but it was still cost-effective given a willingness-to-pay threshold of R$17 586/QALY. Our results showing that the vaccination campaigns in Brazil were cost-saving are consistent with this Brazilian study because, according to our data, the CoronaVac vaccine was applied to less than 25% of the Brazilian population. Given that our analysis contemplates a weighted average of the effectiveness and costs of all the vaccines given in Brazil, the wider use of cost-saving vaccines probably dominated the results in our aggregate country findings.

We believe that our results are conservative estimates because we decided not to include a wider range of relevant costs sometimes known as “indirect costs of illness” (i.e., productivity losses of patients, family members and caregivers). Two of cost-effectiveness studies included indirect costs of illness in their analysis. Wang et al. [12] considered indirect costs of illness due to loss of labor productivity secondary to hospitalizations, secondary to receiving the vaccine, and the possible adverse effect of the vaccine. In addition, Jiang et al. [11] considered indirect costs of illness due to lost work productivity among those infected and for premature death before retirement. Another conservative decision was not considering a “utility benefit” in the vaccinated subjects included in a previous study [6]. Adverse events were not included, as in most previous studies [5–7], as they were usually judged to be neither highly prevalent nor costly nor severe.

Our study has some limitations. As in most economic evaluations published to date, Sars-CoV-2 variants were not explicitly modeled, and results show health outcomes, costs and cost-effectiveness ratios of vaccines previous to the omicron variant and beyond. Also, similarly to previous cost-effectiveness studies, some parameters were not taken from real-life studies but from pivotal randomized trials, oftentimes not performed in the target study countries. As our study focused on the cost-effectiveness of the 2021 vaccination campaigns in this set of countries, the costs and the effects of the booster doses recently incorporated in national campaigns were not included and should be addressed in future studies. Another area where our study could not shed light—a limitation shared by all economic evaluations we reviewed-, and which is important not only in the healthcare sector but in society, is the distributional or equity effects and costs associated with vaccination. Also, many studies have recently described the waning effect of Covid-19 vaccines [37, 38]. At the time of this analysis, evidence was inconclusive, so we assumed (in line with most other economic evaluations [5–8]) a vaccination immunity time of 360 days for the base case scenarios. Our results proved to be robust to a 25% shorter protection period (270 days of immunity from vaccination), when we ran the sensitivity analysis. Finally, we did not include children and adolescents in the study analysis because the start of the vaccination campaign for this population was in the last quarter of 2021, and targeted adults. Future studies should evaluate more recent variants, incremental protection of booster doses, and new information on the waning effect of vaccines.

In summary, cost-effectiveness analysis plays a fundamental role in decision making and the implementation, evaluation, and monitoring of public policies. Our open access and user friendly epidemiological and economic model to assess the impact of vaccination strategies and public health and social measures against COVID-19 constitutes a tool that articulates scientific knowledge, empirical evidence, and public policies in a friendly framework for interaction with users, analysts, and decision-makers. To conclude, the Covid-19 vaccination campaigns have shown to be health beneficial and cost-saving or highly cost-effective in seven countries in Latin American and the Caribbean.

## Supplementary Information

**Additional file 1.** Effectiveness/efficacy of COVID-19 vaccines, COVID-19 vaccines applied in each country, Literature search on epidemiological parameters, Transmission dynamic parameters, General population baseline utility values per-country, Disutility value per disease state, Economic evaluation disaggregated results by country, Deterministic sensitivity analysis, Model calibration, Interactive online CEA model, Advisory board, References.

## Data Availability

The interactive SVEIR model (only Spanish version) is available at: https://www.iecs.org.ar/modelocovid/.
