# Publisher Correction: cost-effectiveness of COVID-19 vaccination in Latin America and the Caribbean: an analysis in Argentina, Brazil, Chile, Colombia, Costa Rica, Mexico, and Peru

**DOI:** 10.1186/s12962-023-00466-4

**Published:** 2023-08-24

**Authors:** Federico Augustovski, Ariel Bardach, Adrián Santoro, Federico Rodriguez-Cairoli, Alejandro López-Osornio, Fernando Argento, Maissa Havela, Alejandro Blumenfeld, Jamile Ballivian, Germán Solioz, Analía Capula, Analía López, Cintia Cejas, William Savedoff, Alfredo Palacios, Adolfo Rubinstein, Andrés Pichon-Riviere

**Affiliations:** 1 Departamento de Evaluación de Tecnologías Sanitarias y Economía de la Salud/Health Technology Assessment and Health Economics Department, Institute for Clinical Effectiveness and Health Policy, Instituto de Efectividad Clínica y Sanitaria (IECS) - CONICET, Dr. Emilio Ravignani 2024 (C1014CPV), Buenos Aires, Argentina; 2 Centro de Implementación e Innovación en Políticas de Salud (CIIPS). Instituto de Efectividad Clínica y Sanitaria (IECS)/Institute for Clinical Effectiveness and Health Policy, Buenos Aires, Argentina; 3 Social Insight, Arrowsic, ME USA

**Cost Effectiveness and Resource Allocation (2023) 21:21**

10.1186/s12962-023-00430-2

Following publication of the original article, the authors flagged the respective given and family names of the authors had been erroneously swapped. The author list has been corrected in the published article and the corrected names can be seen in the author list of this erratum.

The publisher thanks you for reading this erratum and apologizes for any inconvenience caused.

