# Supplementary material for: Cost-effectiveness of COVID-19 vaccination in Latin America and the Caribbean: an analysis in Argentina, Brazil, Chile, Colombia, Costa Rica, Mexico, and Peru
Source: Cost Eff Resour Alloc. 2023 Apr 1;21:21. doi: 10.1186/s12962-023-00430-2 (PMC10066967; doi:10.1186/s12962-023-00430-2)
Supplement: Supplementary file 1 — Additional file 1. Effectiveness/efficacy of COVID-19 vaccines, COVID-19 vaccines applied in each country, Literature search on epidemiological parameters, Transmission dynamic parameters, General population baseline utility values per-country, Disutility value per disease state, Economic evaluation disaggregated results by country, Deterministic sensitivity analysis, Model calibration, Interactive online CEA model, Advisory board, References. [file 12962_2023_430_MOESM1_ESM.docx]

***Supplementary Material***

Effectiveness/efficacy of COVID-19 vaccines

Table 1 summarizes the effectiveness or efficacy value of each COVID-19 vaccine given in at least one of the countries of interest. We report effectiveness or efficacy values per outcome and the number of doses administered. Real-world data was prioritized. If there were no effectiveness data, efficacy data were obtained. In the event that there has been more than one study addressing the effectiveness or efficacy of a particular vaccine, the study with the best methodological approach and/or with a greater number of patients analyzed was chosen.

***Table 1****.* ***Effectiveness/efficacy of each COVID-19 vaccine given in the seven countries of interest***

| ***Vaccine*** | **Doses** | **Effectiveness against symptomatic disease (%)** | **Effectiveness against hospitalizations (%)** | **Effectiveness against ICU admissions (%)** | **Effectiveness against deaths (%)** | **Source** |
| --- | --- | --- | --- | --- | --- | --- |
| ***Gam-COVID-Vac*** | 1 dose | 78.6 | 87.6 | 100.0 | 84.8 | [[1]](https://paperpile.com/c/0ylhSY/pURy) |
|  | 2 doses | 91.6 | 97.7 | 100.0 | 99.9 | [[2]](https://paperpile.com/c/0ylhSY/HBZ6) |
| ***BBIBP-CorV*** | 1 dose | 65.5 | NR | NR | NR | [[3]](https://paperpile.com/c/0ylhSY/wtKK) |
|  | 2 doses | 78.0 | 93.6 | 99.4 | 99.5 | [[4]](https://paperpile.com/c/0ylhSY/HwyU) |
| ***Coronavac*** | 1 dose | 57.9 | 37.4 | 44.7 | 45.7 | [[5]](https://paperpile.com/c/0ylhSY/02Nv) |
|  | 2 doses | 65.9 | 87.5 | 90.3 | 86.3 | [[6]](https://paperpile.com/c/0ylhSY/wnnD) |
| ***BNT162b2/COMIRNATY*** | 1 dose | 82.0 | 74.5 | 77.3 | 71.7 | [[7]](https://paperpile.com/c/0ylhSY/vb2z) |
|  | 2 doses | 97.0 | 97.2 | 97.5 | 96.7 | [[8]](https://paperpile.com/c/0ylhSY/2zPV) |
| ***ChAdOx1_nCoV-19*** | 1 dose | 76.0 | 88.0 | NR | NR | [[9]](https://paperpile.com/c/0ylhSY/MXWs) |
|  | 2 doses | 66.7 | 98.5 | 99.9 | 99.9 | [[9]](https://paperpile.com/c/0ylhSY/MXWs) |
| ***mRNA-1273*** | 1 dose | 93.0 | NR | NR | NR | [[10]](https://paperpile.com/c/0ylhSY/DLu0) |
|  | 2 doses | 94.1 | 95.0 | 93.3 | 93.7 | [[10]](https://paperpile.com/c/0ylhSY/DLu0) |
| ***Ad5-nCoV*** | 1 dose | 74.8 | NR | NR | NR | [[11]](https://paperpile.com/c/0ylhSY/Mn4V) |
| ***Ad26.COV2.S*** | 1 dose | 66.9 | 93.1 | 76.7 | 100.0 | [[12]](https://paperpile.com/c/0ylhSY/aWCR) |

COVID-19 vaccines applied in each country

We used information on all the vaccines purchased or acquired by each country as a proxy for the percentage of each vaccine given in each of the seven countries.

At the date of the analysis, in Argentina, Chile, Costa Rica and Colombia, the contracts for the acquisition of vaccines were confidential, which is why the number of doses acquired was constructed from official government communications and/or media reports[[13]](https://paperpile.com/c/0ylhSY/0Mbr)[[14]](https://paperpile.com/c/0ylhSY/FkRi)[[15]](https://paperpile.com/c/0ylhSY/eiLw)[[16]](https://paperpile.com/c/0ylhSY/9v4C). Brazil (Ministry of Health) and Mexico (Secretary of Foreign Relations, Of. Transparency) presents a public dashboard with information on the acquisition of vaccines by laboratory, detailing the quantities[[17]](https://paperpile.com/c/0ylhSY/QExb)[[18]](https://paperpile.com/c/0ylhSY/zTet). Peru (Ministry of Health) has a summary of contracts made by laboratory and number of vaccines[[19]](https://paperpile.com/c/0ylhSY/YY9o).

***Table 2. Percentage of COVID-19 vaccines applied per country***

|  | ***Argentina*** | ***Brasil*** | ***Chile*** | ***Colombia*** | ***México*** | ***Perú*** | ***Costa Rica*** |
| --- | --- | --- | --- | --- | --- | --- | --- |
| ***ChAdOx1_nCoV-19 (%)*** | 30.5 | 37.4 | 4.9 | 14.1 | 42.7 | 8.2 | 15.8 |
| ***BBIBP-CorV (%)*** | 32.5 | 0 | 0 | 0 | 0 | 33.9 | 0 |
| ***Gam-COVID-Vac (%)*** | 27.6 | 1.6 | 0 | 0 | 11.2 | 0 | 0 |
| ***Ad26.COV2.S (%)*** | 0 | 6.2 | 0 | 8.0 | 0.8 | 0 | 0 |
| ***mRNA-1273 (%)*** | 5.5 | 0 | 0 | 10.9 | 2.0 | 0 | 0 |
| ***BNT162b2/COMIRNATY (%)*** | 3.7 | 32.9 | 18.3 | 33.7 | 23.2 | 57.8 | 84.2 |
| ***Coronavac (%)*** | 0 | 21.9 | 69.2 | 33.1 | 11.7 | 0 | 0 |
| ***Ad5-nCoV (%)*** | 0.2 | 0 | 7.6 | 0 | 8.3 | 0 | 0 |

Literature search on epidemiological parameters

In October 2021 literature search strategies were carried out to ascertain Latinamerican countries COVID-19 specific epidemiological parameters. The main strategies are described below.

***i.*** ***Pubmed:***

*(Americas[Majr] OR Latin America[Mesh] OR America*[tiab] OR Latin America*[all] OR Latinamerica*[all] OR Latinoamerica*[all] OR Latin*[all] OR Hispanic America*[all] OR Hispanoamerica*[all] OR Iberoamerica*[all] OR Ibero Americ*[all] OR Panamerican*[all] OR Central America[Mesh] OR Central America*[tiab] OR Centroamerica*[all] OR Central America[Mesh] OR Central America*[all] OR Centroamerica*[all] OR Mesoamerica*[all] OR Meso America*[all] OR Middle America*[all] OR South America[Mesh] OR South America*[all] OR Southamerica*[all] OR Sudamerica*[all] OR America del sur[all] OR Caribbean Region[Mesh] OR Caribbean[all] OR Caribe*[all] OR West Indies[Mesh] OR West Indi*[all] OR Antill*[all] OR American Native Continental Ancestry Group[Mesh] OR Amerindian*[all] OR Indians[all] OR American Indian*[all] OR Patagoni*[all] OR Andes[all] OR Andean*[all] OR Amazon*[all] OR Argentina[Mesh] OR Argentin*[ad] OR Argentin*[all] OR Argentina[pl] OR Bolivia[Mesh] OR Bolivia*[ad] OR Bolivia*[all] OR Bolivia[pl] OR Colombia[Mesh] OR Colombia*[ad] OR Colombia*[all] OR Colombia[pl] OR Chile[Mesh] OR Chile*[ad] OR Chile*[all] OR Chile[pl] OR Ecuador[Mesh] OR Ecuador*[ad] OR Ecuador*[all] OR Ecuador[pl] OR French Guiana[Mesh] OR Guiana*[ad] OR Guiana*[all] OR French Guiana[pl] OR Guyana[Mesh] OR Guyan*[ad] OR Guyan*[all] OR Guyana[pl] OR Paraguay[Mesh] OR Paraguay*[ad] OR Paraguay*[all] OR Paraguay[pl] OR Peru[Mesh] OR Peru*[ad] OR Peru*[all] OR Peru[pl] OR Suriname[Mesh] OR Surinam*[ad] OR Surinam*[all] OR Suriname[pl] OR Uruguay[Mesh] OR Uruguay*[ad] OR Uruguay*[all] OR Uruguay[pl] OR Venezuela[Mesh] OR Venez*[ad] OR Venez*[all] OR Venezuela[pl] OR Belize[Mesh] OR Belize*[ad] OR Belize*[all] OR Belize[pl] OR Costa Rica[Mesh] OR Costa Ric*[ad] OR Costarric*[ad] OR Costaric*[ad] OR Costa Ric*[all] OR Costarric*[all] OR Costaric*[all] Costa Rica[pl] OR El Salvador[Mesh] OR Salvador*[ad] OR Salvador*[all] OR El Salvador[pl] OR Guatemala[Mesh] OR Guatemal*[ad] OR Guatemal*[all] OR Guatemala[pl] OR Honduras[Mesh] OR Hondur*[ad] OR Hondur*[all] OR Honduras[pl] OR Nicaragua[Mesh] OR Nicaragu*[ad] OR Nicaragu*[all] OR Nicaragua[pl] OR Panama[Mesh] OR Panam*[ad] OR Panam*[all] OR Panama[pl] OR Mexico[Mesh] OR Mexic*[ad] OR Mexic*[all] OR Mejic*[all] OR Mexico[pl] OR Cuba[Mesh] OR Cuba*[ad] OR Cuba*[all] OR Cuba[pl] OR Dominican Republic[Mesh] OR Dominic*[ad] OR Dominic*[all] OR Dominican Republic[pl] OR Haiti[Mesh] OR Haiti*[ad] OR Haiti*[all] OR Haiti[pl] OR Jamaica[Mesh] OR Jamaic*[ad] OR Jamaic*[all] OR Jamaica[pl] OR Puerto Rico[Mesh] OR Puerto Ric*[all] OR Puertorric*[all] OR Puertoric*[all])*

*AND*

*(MH Coronavirus OR MH Coronavirus Infections OR Corona OR COVID-19* OR COVID19* OR 2019-nCoV OR SARS-CoV-2 OR SARS-CoV2 OR SARSCoV2 OR Coronavir* OR Coronovir* OR HCov* OR CV19* OR CV-19* OR N-Cov*) AND (MH Inpatients OR MH Intensive Care Units OR MH Critical Care OR Intensiv* OR Critic* OR Interna* OR ICU) AND (MH Mortality OR Mortalit* OR Mortalidad* OR Death OR Muerte OR Morte)*

*AND (2019/10/01:2021/10/31[pdat])*

### ***ii.*** ***LILACS***

*(MH Coronavirus OR MH Coronavirus Infections OR Corona OR COVID-19$ OR COVID19$ OR 2019-nCoV OR SARS-CoV-2 OR SARS-CoV2 OR SARSCoV2 OR Coronavir$ OR Coronovir$ OR HCov$ OR CV19$ OR CV-19$ OR N-Cov$) AND (MH Inpatients OR MH Intensive Care Units OR MH Critical Care OR Intensiv$ OR Critic$ OR Interna$ OR ICU) AND (MH Mortality OR Mortalit$ OR Mortalidad$ OR Death OR Muerte OR Morte)*

*
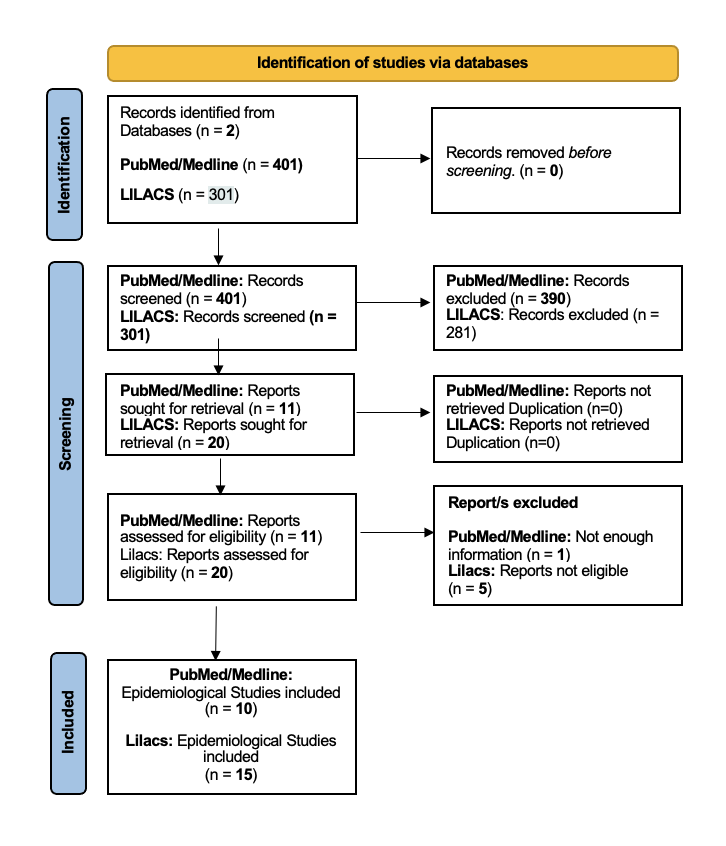
*

Transmission dynamic parameters

Regarding disease transmission dynamics, the model establishes the number of social infections through different transmission probability values according to age groups using contact and an effective contact matrix.

The contact matrix used was based on Prem et al.[[20]](https://paperpile.com/c/0ylhSY/tRTk), which establishes the contacts per-country before the pandemic in different settings and age groups. We used susceptibility and infectiousness data to develop an effective contact matrix for each age group. The interaction of these two matrices generates the expected number of cases through transmission probability values per age group. Measures that reduce contacts (vaccination or public health policies) therefore have an impact on one of these matrices, either by reducing the number of people contacts (for example, school closures) or their effectiveness in transmitting the virus (for instance, vaccination campaign)[[21]](https://paperpile.com/c/0ylhSY/95fg).

Box 1. Covid-19 Hospitalization rates per age group.

| **COVID-19 Hospitalization rate on general ward per-100,000 by age group** |
| --- |
| *18-29: 3.6* |
| *30-39: 5.4* |
| *40-49: 8.5* |
| *50-59: 25.8* |
| *60-69: 44.2* |
| *70-79: 99.2* |
| *80+: 205.6* |
| **COVID-19 Hospitalization rate on ICU per-100,000 by age group** |
| *18-29: 1* |
| *30-39: 1.4* |
| *40-49: 3.5* |
| *50-59: 13.3* |
| *60-69: 24.7* |
| *70-79: 60.8* |
| *80+: 94.4* |

ICU: intensive care unit. Source: Lapidus et al. [[22]](https://paperpile.com/c/0ylhSY/CiI6)

General population baseline utility values per-country

The search strategy that was implemented used the terms "population health utility" together with the term "international" or with the name of each of the countries involved in this study, running a total of eight different searches (one for each country, and one with the term “international”). These searches were carried out during the month of September-October 2021 on Google, prioritizing the first three pages of results obtained.

A total of three sources that report data of interest for Argentina, Brazil, and Colombia were selected.[[23]](https://paperpile.com/c/0ylhSY/vLT1)[[24]](https://paperpile.com/c/0ylhSY/lSuO)[[25]](https://paperpile.com/c/0ylhSY/56wJ)

Given the lack of information for children and young people (group from 0 to 17 years old) for all the sources mentioned, we assumed the same utility value for the following age group (18 to 24 or 29 years old) in all cases. Also, these sources did not report information for the oldest age groups (60-69 years, 70-79 years, and over 80 years) for the case of Brazil and Colombia, but yes for Argentina.

Therefore, the same proportional relationship was applied between the age group 50 to 59 years with the subsequent age groups that were observed in Argentina, extrapolated to the data from Brazil and Colombia, thus obtaining utility values ​​for said age groups in the case of these last two countries.

Given the lack of information for Mexico, Peru, and Chile, it is assumed that the average values ​​of profits are recorded according to the sources available mentioned (average values ​​by age group according to information reported for Argentina, Brazil, and Colombia). For the specific case of Costa Rica, also given the lack of information on baseline utility values ​​after running the search, it was decided to extrapolate information from studies conducted in other Caribbean countries (Barbados, Jamaica, and Trinidad & Tobago).[[26]](https://paperpile.com/c/0ylhSY/Xzt1) [[27]](https://paperpile.com/c/0ylhSY/FK02)

Tables 3 to 7 summarize the baseline utility values ​​for the countries in question, the respective assumptions, and the corresponding bibliographic citations.

**Table 3. Baseline population utility values for each age group for Argentina**

| **Age group** | **Utility value (TTO)** |
| --- | --- |
| 0-17 | 0.951 |
| 18-29 | 0.951 |
| 30-39 | 0.936 |
| 40-49 | 0.919 |
| 50-59 | 0.898 |
| 60-69 | 0.874 |
| 70-79 | 0.835 |
| >80 | 0.756 |

**Table 4. Baseline population utility values for each age group for Brazil**

| **Age group** | **Utility value (TTO)** |
| --- | --- |
| 0-17 | 0.978 |
| 18-29 | 0.978 |
| 30-39 | 0.970 |
| 40-49 | 0.930 |
| 50-59 | 0.930 |
| 60-69 | 0.905 |
| 70-79 | 0.865 |

**Table 5. Baseline population utility values for each age group for Colombia**

| **Age group** | **Utility value (TTO)** |
| --- | --- |
| 0-17 | 0.970 |
| 18-29 | 0.970 |
| 30-39 | 0.962 |
| 40-49 | 0.957 |
| 50-59 | 0.939 |
| 60-69 | 0.914 |
| 70-79 | 0.873 |
| >80 | 0.791 |

**Table 6. Baseline population utility values for each age group for Chile, México, and Peru**

| **Age group** | **Utility value (TTO)** |
| --- | --- |
| 0-17 | 0.966 |
| 18-29 | 0.966 |
| 30-39 | 0.956 |
| 40-49 | 0.935 |
| 50-59 | 0.922 |
| 60-69 | 0.898 |
| 70-79 | 0.858 |
| >80 | 0.776 |

**Table 7. Baseline population utility values for each age group for Costa Rica.**

| **Age group** | **Utility value (TTO)** |
| --- | --- |
| 0-17 | 0.965 |
| 18-29 | 0.965 |
| 30-39 | 0.964 |
| 40-49 | 0.953 |
| 50-59 | 0.940 |
| 60-69 | 0.919 |
| >70 | 0.884 |

Disutility value per disease state - search strategy

Recently published studies of the cost-effectiveness analysis of vaccines for Covid-19 were reviewed using the terms "cost-effectiveness" and "covid vaccine." The search was carried out during September 2021 in PubMed, and in gray literature (Google search engine), prioritizing the first three pages of results obtained. Additionally, specific utility/disutility values for each health state were incorporated. Among all the economic evaluations for Covid-19 vaccines identified, four of them report quality-adjusted life years lost.[[28–31]](https://paperpile.com/c/0ylhSY/2tXpH+ZQK4A+Nyll4+raY0e)

In particular, the Kohli 2021 study performs a cost-effectiveness analysis secondary to the implementation of the vaccination strategy against Covid-19 in the United States, using different dis-utility values ​​for the following states: symptomatic infected patient, hospitalized infected patient, hospitalized infected patient with the critical unit requirement, infected patient with ICU requirement and invasive mechanical ventilation.[[31]](https://paperpile.com/c/0ylhSY/raY0e) These disutility data were obtained mainly from economic models recently developed by the United States ICER agency to evaluate treatments against Covid-19, and from older studies, in the context of Influenza virus infection and Clostridium Difficile hospitalizations. On the other hand, the Padula 2021 study proposes, as an assumption of its model, that non-infected subjects who manage to be vaccinated present a 2% gain in utility value compared to their baseline utility value, which represents a preference of the individual to be vaccinated.[[30]](https://paperpile.com/c/0ylhSY/Nyll4) Our model is conservative in that regard and does not incorporate this assumption of improved long-term utility by being vaccinated. As a result of the uncertainty and little available evidence about the quality of life of an average subject who has experienced Covid-19 infection but still has sequelae of said disease (known as Long Covid or Post Covid), it was decided do not to enter data from different utilities for the status of “Recovered” in the model. Most of the reviewed and mentioned models agreed and chose to implement the same assumption.

Economic evaluation disaggregated results

In this section we report disaggregated results for each of the countries, including years of life lost, total cases and their costs, symptomatic cases and their costs, general ward/intensive care unit hospitalizations and their costs, and costs of vaccination campaigns. Undiscounted and discounted results are presented as well as discounted results where applicable. Costs are expressed in American dollars for November 2021.

***Table 8. Disaggregated results for Argentina - Standard Vaccination Campaign results***

| ***Outcome*** | ***No Vaccination*** | ***Standard Vaccination Campaign*** | ***Difference*** |
| --- | --- | --- | --- |
| *Total costs ($)* | *2,489,103,233* | *2,244,620,488* | *(244,482,745)* |
| *QALYs*  *lost (3% annual disc. rate)* | *1,678,317* | *1,098,725* | *(579,592)* |
| *Total COVID-19 cases* | *26,282,807* | *21,255,577* | *(5,027,230)* |
| *Hospitalization days on general ward* | *1,181,688* | *751,685* | *(430,003)* |
| *Hospitalization days on ICU* | *2,542,614* | *1,550,926* | *(991,688)* |
| *Total deaths* | *160,172* | *93,013* | *(67,159)* |
| *Years of life lost (3% annual disc. rate)* | *2,029,168* | *1,321,194* | *(707,974)* |
| *Years of life lost (without disc. rate)* | *2,860,409* | *1,957,545* | *(902,864)* |
| *QALY lost (without disc. rate)* | *2,408,228* | *1,661,205* | *(747,023)* |
| *Total applied vaccines* | *0* | *43,741,205* | *43,741,205* |
| *Vaccination campaign costs* | *-* | *387,547,076* | *387,547,076* |
| *Total COVID-19 event costs* | *2,489,103,233* | *1,857,073,412* | *(632,029,821)* |
| *Costs of non-hospitalized symptomatic cases* | *1,684,359,973* | *1,362,184,925* | *(322,175,048)* |
| *Hospitalization on general ward costs* | *153,678,568* | *97,756,572* | *(55,921,996)* |
| *Hospitalization on ICU costs (without requirement of mechanical ventilation)* | *176,390,768* | *107,593,614* | *(68,797,154)* |
| *Hospitalization on ICU costs (with mechanical ventilation)* | *474,673,924* | *289,538,300* | *(185,135,624)* |
|  |  |  |  |
| ***Table 9. Disaggregated results for Argentina - Optimized Vaccination Campaign results*** | | | |
|  |  |  |  |
| ***Outcome*** | ***No Vaccination*** | ***Optimized Vaccination Campaign*** | ***Difference*** |
| *Total costs ($)* | *2,489,103,233* | *2,159,588,975* | *(329,514,258)* |
| *QALYs*  *lost (3% annual disc. rate)* | *1,678,317* | *868,024* | *(810,293)* |
| *Total COVID-19 cases* | *26,282,807* | *18,996,228* | *(7,286,579)* |
| *Hospitalization days on general ward* | *1,181,688* | *569,011* | *(612,677)* |
| *Hospitalization days on ICU* | *2,542,614* | *1,120,343* | *(1,422,271)* |
| *Total deaths* | *160,172* | *66,227* | *(93,945)* |
| *Years of life lost (3% annual disc. rate)* | *2,029,168* | *1,039,462* | *(989,706)* |
| *Years of life lost (without disc. rate)* | *2,860,409* | *1,597,516* | *(1,262,893)* |
| *QALY lost (without disc. rate)* | *2,408,228* | *1,363,078* | *(1,045,150)* |
| *Total applied vaccines* | *0* | *65,611,807* | *65,611,807* |
| *Vaccination campaign costs* | *-* | *581,320,614* | *581,320,614* |
| *Total COVID-19 event costs* | *2,489,103,233* | *1,578,268,362* | *(910,834,871)* |
| *Costs of non-hospitalized symptomatic cases* | *1,684,359,973* | *1,217,392,236* | *(466,967,737)* |
| *Hospitalization on general ward costs* | *153,678,568* | *73,999,821* | *(79,678,747)* |
| *Hospitalization on ICU costs (without requirement of mechanical ventilation)* | *176,390,768* | *77,722,433* | *(98,668,335)* |
| *Hospitalization on ICU costs (with mechanical ventilation)* | *474,673,924* | *209,153,872* | *(265,520,052)* |
|  |  |  |  |
| ***Table 10. Disaggregated results for Argentina – Realistic Vaccination Campaign results*** | | | |
| ***Outcome*** | ***No Vaccination*** | ***Realistic Vaccination Campaign*** | ***Difference*** |
| *Total costs ($)* | *2,489,103,233* | *2,222,348,451* | *(266,754,782)* |
| *QALYs*  *lost (3% annual disc. rate)* | *1,678,317* | *940,879* | *(737,438)* |
| *Total COVID-19 cases* | *26,282,807* | *20,144,558* | *(6,138,249)* |
| *Hospitalization days on general ward* | *1,181,688* | *630,461* | *(551,227)* |
| *Hospitalization days on ICU* | *2,542,614* | *1,251,293* | *(1,291,321)* |
| *Total deaths* | *160,172* | *74,758* | *(85,414)* |
| *Years of life lost (3% annual disc. rate)* | *2,029,168* | *1,128,493* | *(900,675)* |
| *Years of life lost (without disc. rate)* | *2,860,409* | *1,712,234* | *(1,148,175)* |
| *QALY lost (without disc. rate)* | *2,408,228* | *1,458,187* | *(950,041)* |
| *Total applied vaccines* | *0* | *58,904,823* | *58,904,823* |
| *Vaccination campaign costs* | *-* | *528,965,308* | *528,965,308* |
| *Total COVID-19 event costs* | *2,489,103,233* | *1,693,383,143* | *(795,720,090)* |
| *Costs of non-hospitalized symptomatic cases* | *1,684,359,973* | *1,290,984,117* | *(393,375,856)* |
| *Hospitalization on general ward costs* | *153,678,568* | *81,991,392* | *(71,687,176)* |
| *Hospitalization on ICU costs (without requirement of mechanical ventilation)* | *176,390,768* | *86,806,963* | *(89,583,805)* |
| *Hospitalization on ICU costs (with mechanical ventilation)* | *474,673,924* | *233,600,671* | *(241,073,253)* |

***Table 11. Disaggregated results for Brazil - Standard Vaccination Campaign results***

| ***Outcome*** | ***No Vaccination*** | ***Standard Vaccination Campaign*** | ***Difference*** |
| --- | --- | --- | --- |
| *Total costs ($)* | *14,899,000,000* | *10,248,805,980* | *(4,650,194,020)* |
| *QALYs*  *lost (3% annual disc. rate)* | *3,650,818* | *2,549,322* | *(1,101,496)* |
| *Total COVID-19 cases* | *204,887,970* | *112,188,246* | *(92,699,724)* |
| *Hospitalization days on general ward* | *6,224,955* | *3,645,868* | *(2,579,087)* |
| *Hospitalization days on ICU* | *11,691,124* | *7,464,641* | *(4,226,483)* |
| *Total deaths* | *330,091* | *200,640* | *(129,451)* |
| *Years of life lost (3% annual disc. rate)* | *4,275,760* | *2,968,033* | *(1,307,727)* |
| *Years of life lost (without disc. rate)* | *6,066,539* | *4,401,992* | *(1,664,547)* |
| *QALY lost (without disc. rate)* | *7,153,394* | *5,008,540* | *(2,144,854)* |
| *Total applied vaccines* | *0* | *205,628,827* | *205,628,827* |
| *Vaccination campaign costs* | *-* | *1,821,871,411* | *1,821,871,411* |
| *Total COVID-19 event costs* | *14,899,000,000* | *8,426,934,569* | *(6,472,065,431)* |
| *Costs of non-hospitalized symptomatic cases* | *11,851,000,000* | *6,489,360,827* | *(5,361,639,173)* |
| *Hospitalization on general ward costs* | *161,413,083* | *94,537,361* | *(66,875,722)* |
| *Hospitalization on ICU costs (without requirement of mechanical ventilation)* | *801,428,855* | *511,702,660* | *(289,726,195)* |
| *Hospitalization on ICU costs (with mechanical ventilation)* | *2,085,135,260* | *1,331,333,721* | *(753,801,539)* |

***Table 12. Disaggregated results for Brazil - Optimized Vaccination Campaign results***

| **Outcome** | **No Vaccination** | **Optimized Vaccination Campaign** | **Difference** |
| --- | --- | --- | --- |
| Total costs ($) | 14,899,000,000 | 10,410,709,554 | (4,488,290,446) |
| QALYs  lost (3% annual disc. rate) | 3,650,818 | 2,231,441 | (1,419,377) |
| Total COVID-19 cases | 204,887,970 | 105,342,474 | (99,545,496) |
| Hospitalization days on general ward | 6,224,955 | 3,067,007 | (3,157,948) |
| Hospitalization days on ICU | 11,691,124 | 6,095,521 | (5,595,603) |
| Total deaths | 330,091 | 159,823 | (170,268) |
| Years of life lost (3% annual disc. rate) | 4,275,760 | 2,588,404 | (1,687,356) |
| Years of life lost (without disc. rate) | 6,066,539 | 3,937,047 | (2,129,492) |
| QALY lost (without disc. rate) | 7,153,394 | 4,398,674 | (2,754,720) |
| Total applied vaccines | 0 | 308,443,241 | 308,443,241 |
| Vaccination campaign costs | - | 2,732,807,116 | 2,732,807,116 |
| Total COVID-19 event costs | 14,899,000,000 | 7,677,902,438 | (7,221,097,562) |
| Costs of non-hospitalized symptomatic cases | 11,851,000,000 | 6,093,377,368 | (5,757,622,632) |
| Hospitalization on general ward costs | 161,413,083 | 79,527,484 | (81,885,599) |
| Hospitalization on ICU costs (without requirement of mechanical ventilation) | 801,428,855 | 417,849,195 | (383,579,660) |
| Hospitalization on ICU costs (with mechanical ventilation) | 2,085,135,260 | 1,087,148,391 | (997,986,869) |

***Table 13. Disaggregated results for Brazil - Realistic Vaccination Campaign results***

| ***Outcome*** | ***No Vaccination*** | ***Realistic Vaccination Campaign*** | ***Difference*** |
| --- | --- | --- | --- |
| *Total costs ($)* | *14,899,000,000* | *10,298,972,430* | *(4,600,027,570)* |
| *QALYs*  *lost (3% annual disc. rate)* | *3,650,818* | *2,368,674* | *(1,282,144)* |
| *Total COVID-19 cases* | *204,887,970* | *108,871,116* | *(96,016,854)* |
| *Hospitalization days on general ward* | *6,224,955* | *3,338,984* | *(2,885,971)* |
| *Hospitalization days on ICU* | *11,691,124* | *6,754,584* | *(4,936,540)* |
| *Total deaths* | *330,091* | *177,544* | *(152,547)* |
| *Years of life lost (3% annual disc. rate)* | *4,275,760* | *2,752,414* | *(1,523,346)* |
| *Years of life lost (without disc. rate)* | *6,066,539* | *4,138,523* | *(1,928,016)* |
| *QALY lost (without disc. rate)* | *7,153,394* | *4,659,206* | *(2,494,188)* |
| *Total applied vaccines* | *0* | *263,136,356* | *263,136,356* |
| *Vaccination campaign costs* | *-* | *2,247,184,481* | *2,247,184,481* |
| *Total COVID-19 event costs* | *14,899,000,000* | *8,051,787,948* | *(6,847,212,052)* |
| *Costs of non-hospitalized symptomatic cases* | *11,851,000,000* | *6,297,486,398* | *(5,553,513,602)* |
| *Hospitalization on general ward costs* | *161,413,083* | *86,579,859* | *(74,833,224)* |
| *Hospitalization on ICU costs (without requirement of mechanical ventilation)* | *801,428,855* | *463,028,096* | *(338,400,759)* |
| *Hospitalization on ICU costs (with mechanical ventilation)* | *2,085,135,260* | *1,204,693,596* | *(880,441,664)* |

***Table 14. Disaggregated results for Chile - Standard Vaccination Campaign results***

| ***Outcome*** | ***No Vaccination*** | ***Standard Vaccination Campaign*** | ***Difference*** |
| --- | --- | --- | --- |
| *Total costs ($)* | *1,426,693,488* | *1,346,544,316* | *(80,149,172)* |
| *QALYs*  *lost (3% annual disc. rate)* | *423,399* | *315,238* | *(108,161)* |
| *Total COVID-19 cases* | *11,993,286* | *10,461,493* | *(1,531,793)* |
| *Hospitalization days on general ward* | *558,559* | *402,300* | *(156,259)* |
| *Hospitalization days on ICU* | *1,203,916* | *839,742* | *(364,174)* |
| *Total deaths* | *38,672* | *25,379* | *(13,293)* |
| *Years of life lost (3% annual disc. rate)* | *507,735* | *377,125* | *(130,610)* |
| *Years of life lost (without disc. rate)* | *737,847* | *569,437* | *(168,410)* |
| *QALY lost (without disc. rate)* | *613,901* | *474,133* | *(139,768)* |
| *Total applied vaccines* | *0* | *17,729,989* | *17,729,989* |
| *Vaccination campaign costs* | *-* | *157,087,701* | *157,087,701* |
| *Total COVID-19 event costs* | *1,426,693,488* | *1,189,456,615* | *(237,236,873)* |
| *Costs of non-hospitalized symptomatic cases* | *1,100,330,034* | *959,794,909* | *(140,535,125)* |
| *Hospitalization on general ward costs* | *88,855,600* | *63,997,874* | *(24,857,726)* |
| *Hospitalization on ICU costs (without requirement of mechanical ventilation)* | *66,782,639* | *46,581,482* | *(20,201,157)* |
| *Hospitalization on ICU costs (with mechanical ventilation)* | *170,725,214* | *119,082,350* | *(51,642,864)* |

***Table 15. Disaggregated results for Chile - Optimized Vaccination Campaign results***

| ***Outcome*** | ***No Vaccination*** | ***Optimized Vaccination Campaign*** | ***Difference*** |
| --- | --- | --- | --- |
| *Total costs ($)* | *1,426,693,488* | *1,324,404,167* | *(102,289,321)* |
| *QALYs*  *lost (3% annual disc. rate)* | *423,399* | *272,668* | *(150,731)* |
| *Total COVID-19 cases* | *11,993,286* | *9,826,170* | *(2,167,116)* |
| *Hospitalization days on general ward* | *558,559* | *335,069* | *(223,490)* |
| *Hospitalization days on ICU* | *1,203,916* | *679,051* | *(524,865)* |
| *Total deaths* | *38,672* | *20,048* | *(18,624)* |
| *Years of life lost (3% annual disc. rate)* | *507,735* | *325,622* | *(182,113)* |
| *Years of life lost (without disc. rate)* | *737,847* | *503,086* | *(234,761)* |
| *QALY lost (without disc. rate)* | *613,901* | *419,171* | *(194,730)* |
| *Total applied vaccines* | *0* | *26,594,983* | *26,594,983* |
| *Vaccination campaign costs* | *-* | *235,631,551* | *235,631,551* |
| *Total COVID-19 event costs* | *1,426,693,488* | *1,088,772,615* | *(337,920,873)* |
| *Costs of non-hospitalized symptomatic cases* | *1,100,330,034* | *901,506,911* | *(198,823,123)* |
| *Hospitalization on general ward costs* | *88,855,600* | *53,302,839* | *(35,552,761)* |
| *Hospitalization on ICU costs (without requirement of mechanical ventilation)* | *66,782,639* | *37,667,780* | *(29,114,859)* |
| *Hospitalization on ICU costs (with mechanical ventilation)* | *170,725,214* | *96,295,084* | *(74,430,130)* |

***Table 16. Disaggregated results for Chile - Realistic Vaccination Campaign results***

| **Outcome** | **No Vaccination** | **Realistic Vaccination Campaign** | **Difference** |
| --- | --- | --- | --- |
| Total costs ($) | 1,426,693,488 | 1,429,861,357 | 3,167,869 |
| QALYs  lost (3% annual disc. rate) | 423,399 | 279,142 | (144,257) |
| Total COVID-19 cases | 11,993,286 | 10,166,159 | (1,827,127) |
| Hospitalization days on general ward | 558,559 | 356,198 | (202,361) |
| Hospitalization days on ICU | 1,203,916 | 724,862 | (479,054) |
| Total deaths | 38,672 | 20,795 | (17,877) |
| Years of life lost (3% annual disc. rate) | 507,735 | 333,474 | (174,261) |
| Years of life lost (without disc. rate) | 737,847 | 513,786 | (224,061) |
| QALY lost (without disc. rate) | 613,901 | 427,995 | (185,906) |
| Total applied vaccines | 0 | 25,959,659 | 25,959,659 |
| Vaccination campaign costs | - | 297,497,688 | 297,497,688 |
| Total COVID-19 event costs | 1,426,693,488 | 1,132,363,669 | (294,329,819) |
| Costs of non-hospitalized symptomatic cases | 1,100,330,034 | 932,699,306 | (167,630,728) |
| Hospitalization on general ward costs | 88,855,600 | 56,663,912 | (32,191,688) |
| Hospitalization on ICU costs (without requirement of mechanical ventilation) | 66,782,639 | 40,208,975 | (26,573,664) |
| Hospitalization on ICU costs (with mechanical ventilation) | 170,725,214 | 102,791,475 | (67,933,739) |

***Table 17. Disaggregated results for Colombia - Standard Vaccination Campaign results***

| ***Outcome*** | ***No Vaccination*** | ***Standard Vaccination Campaign*** | ***Difference*** |
| --- | --- | --- | --- |
| *Total costs ($)* | *5,213,429,639* | *3,992,977,174* | *(1,220,452,465)* |
| *QALYs*  *lost (3% annual disc. rate)* | *1,878,855* | *1,337,234* | *(541,621)* |
| *Total COVID-19 cases* | *38,379,802* | *26,420,301* | *(11,959,501)* |
| *Hospitalization days on general ward* | *1,360,731* | *895,686* | *(465,045)* |
| *Hospitalization days on ICU* | *2,793,676* | *1,861,663* | *(932,013)* |
| *Total deaths* | *157,566* | *99,594* | *(57,972)* |
| *Years of life lost (3% annual disc. rate)* | *2,206,716* | *1,567,450* | *(639,266)* |
| *Years of life lost (without disc. rate)* | *3,196,138* | *2,378,618* | *(817,520)* |
| *QALY lost (without disc. rate)* | *2,720,486* | *2,026,060* | *(694,426)* |
| *Total applied vaccines* | *0* | *49,411,426* | *49,411,426* |
| *Vaccination campaign costs* | *-* | *437,785,236* | *437,785,236* |
| *Total COVID-19 event costs* | *5,213,429,639* | *3,555,191,938* | *(1,658,237,701)* |
| *Costs of non-hospitalized symptomatic cases* | *3,790,101,430* | *2,609,070,738* | *(1,181,030,692)* |
| *Hospitalization on general ward costs* | *290,094,264* | *190,951,394* | *(99,142,870)* |
| *Hospitalization on ICU costs (without requirement of mechanical ventilation)* | *322,275,934* | *214,759,764* | *(107,516,170)* |
| *Hospitalization on ICU costs (with mechanical ventilation)* | *810,958,010* | *540,410,042* | *(270,547,968)* |

***Table 18. Disaggregated results for Colombia - Optimized Vaccination Campaign results***

| ***Outcome*** | ***No Vaccination*** | ***Optimized Vaccination Campaign*** | ***Difference*** |
| --- | --- | --- | --- |
| *Total costs ($)* | *5,213,429,639* | *3,854,869,299* | *(1,358,560,340)* |
| *QALYs*  *lost (3% annual disc. rate)* | *1,878,855* | *1,136,268* | *(742,587)* |
| *Total COVID-19 cases* | *38,379,802* | *24,734,473* | *(13,645,329)* |
| *Hospitalization days on general ward* | *1,360,731* | *735,256* | *(625,475)* |
| *Hospitalization days on ICU* | *2,793,676* | *1,476,303* | *(1,317,373)* |
| *Total deaths* | *157,566* | *77,416* | *(80,150)* |
| *Years of life lost (3% annual disc. rate)* | *2,206,716* | *1,329,913* | *(876,803)* |
| *Years of life lost (without disc. rate)* | *3,196,138* | *2,078,942* | *(1,117,196)* |
| *QALY lost (without disc. rate)* | *2,720,486* | *1,771,777* | *(948,709)* |
| *Total applied vaccines* | *0* | *74,117,139* | *74,117,139* |
| *Vaccination campaign costs* | *-* | *656,677,854* | *656,677,854* |
| *Total COVID-19 event costs* | *5,213,429,639* | *3,198,191,445* | *(2,015,238,194)* |
| *Costs of non-hospitalized symptomatic cases* | *3,790,101,430* | *2,442,591,035* | *(1,347,510,395)* |
| *Hospitalization on general ward costs* | *290,094,264* | *156,749,196* | *(133,345,068)* |
| *Hospitalization on ICU costs (without requirement of mechanical ventilation)* | *322,275,934* | *170,304,936* | *(151,970,998)* |
| *Hospitalization on ICU costs (with mechanical ventilation)* | *810,958,010* | *428,546,278* | *(382,411,732)* |

***Table 19. Disaggregated results for Colombia- Realistic Vaccination Campaign results***

| **Outcome** | **No Vaccination** | **Realistic Vaccination Campaign** | **Difference** |
| --- | --- | --- | --- |
| Total costs ($) | 5,213,429,639 | 4,270,583,078 | (942,846,561) |
| QALYs  lost (3% annual disc. rate) | 1,878,855 | 1,246,398 | (632,457) |
| Total COVID-19 cases | 38,379,802 | 25,837,530 | (12,542,272) |
| Hospitalization days on general ward | 1,360,731 | 830,364 | (530,367) |
| Hospitalization days on ICU | 2,793,676 | 1,703,976 | (1,089,700) |
| Total deaths | 157,566 | 89,570 | (67,996) |
| Years of life lost (3% annual disc. rate) | 2,206,716 | 1,460,140 | (746,576) |
| Years of life lost (without disc. rate) | 3,196,138 | 2,243,718 | (952,420) |
| QALY lost (without disc. rate) | 2,720,486 | 1,911,528 | (808,958) |
| Total applied vaccines | 0 | 60,471,350 | 60,471,350 |
| Vaccination campaign costs | - | 850,831,900 | 850,831,900 |
| Total COVID-19 event costs | 5,213,429,639 | 3,419,751,177 | (1,793,678,462) |
| Costs of non-hospitalized symptomatic cases | 3,790,101,430 | 2,551,520,634 | (1,238,580,796) |
| Hospitalization on general ward costs | 290,094,264 | 177,025,295 | (113,068,969) |
| Hospitalization on ICU costs (without requirement of mechanical ventilation) | 322,275,934 | 196,569,136 | (125,706,798) |
| Hospitalization on ICU costs (with mechanical ventilation) | 810,958,010 | 494,636,113 | (316,321,897) |

***Table 20. Disaggregated results for Costa Rica - Standard Vaccination Campaign results***

| ***Outcome*** | ***No Vaccination*** | ***Standard Vaccination Campaign*** | ***Difference*** |
| --- | --- | --- | --- |
| *Total costs ($)* | *1,000,387,362* | *681,338,274* | *(319,049,088)* |
| *QALYs*  *lost (3% annual disc. rate)* | *130,323* | *89,120* | *(41,203)* |
| *Total COVID-19 cases* | *3,723,956* | *2,456,502* | *(1,267,454)* |
| *Hospitalization days on general ward* | *128,734* | *81,020* | *(47,714)* |
| *Hospitalization days on ICU* | *261,712* | *167,108* | *(94,604)* |
| *Total deaths* | *10,442* | *6,343* | *(4,099)* |
| *Years of life lost (3% annual disc. rate)* | *146,351* | *100,196* | *(46,155)* |
| *Years of life lost (without disc. rate)* | *219,040* | *156,638* | *(62,402)* |
| *QALY lost (without disc. rate)* | *196,027* | *140,234* | *(55,793)* |
| *Total applied vaccines* | *0* | *4,773,932* | *4,773,932* |
| *Vaccination campaign costs* | *-* | *42,297,034* | *42,297,034* |
| *Total COVID-19 event costs* | *1,000,387,362* | *639,041,240* | *(361,346,122)* |
| *Costs of non-hospitalized symptomatic cases* | *40,963,511* | *27,021,526* | *(13,941,985)* |
| *Hospitalization on general ward costs* | *64,367,219* | *40,510,049* | *(23,857,170)* |
| *Hospitalization on ICU costs (without requirement of mechanical ventilation)* | *151,793,230* | *96,922,692* | *(54,870,538)* |
| *Hospitalization on ICU costs (with mechanical ventilation)* | *743,263,402* | *474,586,974* | *(268,676,428)* |

***Table 21. Disaggregated results for Costa Rica - Optimized Vaccination Campaign results***

| ***Outcome*** | ***No Vaccination*** | ***Optimized Vaccination Campaign*** | ***Difference*** |
| --- | --- | --- | --- |
| *Total costs ($)* | *1,000,387,362* | *559,545,058* | *(440,842,304)* |
| *QALYs*  *lost (3% annual disc. rate)* | *130,323* | *73,883* | *(56,440)* |
| *Total COVID-19 cases* | *3,723,956* | *2,265,327* | *(1,458,629)* |
| *Hospitalization days on general ward* | *128,734* | *64,711* | *(64,023)* |
| *Hospitalization days on ICU* | *261,712* | *128,312* | *(133,400)* |
| *Total deaths* | *10,442* | *4,791* | *(5,651)* |
| *Years of life lost (3% annual disc. rate)* | *146,351* | *83,130* | *(63,221)* |
| *Years of life lost (without disc. rate)* | *219,040* | *133,769* | *(85,271)* |
| *QALY lost (without disc. rate)* | *196,027* | *119,784* | *(76,243)* |
| *Total applied vaccines* | *0* | *7,160,897* | *7,160,897* |
| *Vaccination campaign costs* | *-* | *63,445,551* | *63,445,551* |
| *Total COVID-19 event costs* | *1,000,387,362* | *496,099,508* | *(504,287,854)* |
| *Costs of non-hospitalized symptomatic cases* | *40,963,511* | *24,918,600* | *(16,044,911)* |
| *Hospitalization on general ward costs* | *64,367,219* | *32,355,532* | *(32,011,687)* |
| *Hospitalization on ICU costs (without requirement of mechanical ventilation)* | *151,793,230* | *74,420,678* | *(77,372,552)* |
| *Hospitalization on ICU costs (with mechanical ventilation)* | *743,263,402* | *364,404,698* | *(378,858,704)* |

***Table 22. Disaggregated results for Costa Rica - Realistic Vaccination Campaign results***

| **Outcome** | **No Vaccination** | **Realistic Vaccination Campaign** | **Difference** |
| --- | --- | --- | --- |
| Total costs ($) | 1,000,387,362 | 626,362,313 | (374,025,049) |
| QALYs  lost (3% annual disc. rate) | 130,323 | 80,698 | (49,625) |
| Total COVID-19 cases | 3,723,956 | 2,352,102 | (1,371,854) |
| Hospitalization days on general ward | 128,734 | 72,096 | (56,638) |
| Hospitalization days on ICU | 261,712 | 145,772 | (115,940) |
| Total deaths | 10,442 | 5,487 | (4,955) |
| Years of life lost (3% annual disc. rate) | 146,351 | 90,764 | (55,587) |
| Years of life lost (without disc. rate) | 219,040 | 144,005 | (75,035) |
| QALY lost (without disc. rate) | 196,027 | 128,937 | (67,090) |
| Total applied vaccines | 0 | 6,018,336 | 6,018,336 |
| Vaccination campaign costs | - | 65,900,784 | 65,900,784 |
| Total COVID-19 event costs | 1,000,387,362 | 560,461,529 | (439,925,833) |
| Costs of non-hospitalized symptomatic cases | 40,963,511 | 25,873,124 | (15,090,387) |
| Hospitalization on general ward costs | 64,367,219 | 36,048,103 | (28,319,116) |
| Hospitalization on ICU costs (without requirement of mechanical ventilation) | 151,793,230 | 84,547,770 | (67,245,460) |
| Hospitalization on ICU costs (with mechanical ventilation) | 743,263,402 | 413,992,531 | (329,270,871) |

***Table 23. Disaggregated results for México - Standard Vaccination Campaign results***

| ***Outcome*** | ***No Vaccination*** | ***Standard Vaccination Campaign*** | ***Difference*** |
| --- | --- | --- | --- |
| *Total costs ($)* | *24,460,665,837* | *15,168,858,043* | *(9,291,807,794)* |
| *QALYs*  *lost (3% annual disc. rate)* | *4,304,472* | *2,784,670* | *(1,519,802)* |
| *Total COVID-19 cases* | *108,790,250* | *59,200,569* | *(49,589,681)* |
| *Hospitalization days on general ward* | *3,214,401* | *1,777,950* | *(1,436,451)* |
| *Hospitalization days on ICU* | *6,107,399* | *3,640,301* | *(2,467,098)* |
| *Total deaths* | *385,521* | *219,716* | *(165,805)* |
| *Years of life lost (3% annual disc. rate)* | *5,072,737* | *3,262,947* | *(1,809,790)* |
| *Years of life lost (without disc. rate)* | *7,193,633* | *4,872,228* | *(2,321,405)* |
| *QALY lost (without disc. rate)* | *6,210,449* | *4,238,984* | *(1,971,465)* |
| *Total applied vaccines* | *0* | *126,194,571* | *126,194,571* |
| *Vaccination campaign costs* | *-* | *1,118,083,900* | *1,118,083,900* |
| *Total COVID-19 event costs* | *24,460,665,837* | *14,050,774,143* | *(10,409,891,694)* |
| *Costs of non-hospitalized symptomatic cases* | *9,014,088,128* | *4,905,211,118* | *(4,108,877,010)* |
| *Hospitalization on general ward costs* | *1,428,769,108* | *790,281,182* | *(638,487,926)* |
| *Hospitalization on ICU costs (without requirement of mechanical ventilation)* | *3,748,736,367* | *2,234,425,493* | *(1,514,310,874)* |
| *Hospitalization on ICU costs (with mechanical ventilation)* | *10,269,072,234* | *6,120,856,350* | *(4,148,215,884)* |

***Table 24. Disaggregated results for México - Optimized Vaccination Campaign results***

| ***Outcome*** | ***No Vaccination*** | ***Optimized Vaccination Campaign*** | ***Difference*** |
| --- | --- | --- | --- |
| *Total costs ($)* | *24,460,665,837* | *13,336,807,403* | *(11,123,858,434)* |
| *QALYs*  *lost (3% annual disc. rate)* | *4,304,472* | *2,332,495* | *(1,971,977)* |
| *Total COVID-19 cases* | *108,790,250* | *54,743,719* | *(54,046,531)* |
| *Hospitalization days on general ward* | *3,214,401* | *1,432,471* | *(1,781,930)* |
| *Hospitalization days on ICU* | *6,107,399* | *2,826,327* | *(3,281,072)* |
| *Total deaths* | *385,521* | *167,396* | *(218,125)* |
| *Years of life lost (3% annual disc. rate)* | *5,072,737* | *2,722,691* | *(2,350,046)* |
| *Years of life lost (without disc. rate)* | *7,193,633* | *4,199,666* | *(2,993,967)* |
| *QALY lost (without disc. rate)* | *6,210,449* | *3,670,709* | *(2,539,740)* |
| *Total applied vaccines* | *0* | *189,291,857* | *189,291,857* |
| *Vaccination campaign costs* | *-* | *1,677,125,850* | *1,677,125,850* |
| *Total COVID-19 event costs* | *24,460,665,837* | *11,659,681,553* | *(12,800,984,284)* |
| *Costs of non-hospitalized symptomatic cases* | *9,014,088,128* | *4,535,927,707* | *(4,478,160,421)* |
| *Hospitalization on general ward costs* | *1,428,769,108* | *636,719,055* | *(792,050,053)* |
| *Hospitalization on ICU costs (without requirement of mechanical ventilation)* | *3,748,736,367* | *1,734,806,340* | *(2,013,930,027)* |
| *Hospitalization on ICU costs (with mechanical ventilation)* | *10,269,072,234* | *4,752,228,451* | *(5,516,843,783)* |

***Table 25. Disaggregated results for México - Realistic Vaccination Campaign results***

| ***Outcome*** | ***No Vaccination*** | ***Realistic Vaccination Campaign*** | ***Difference*** |
| --- | --- | --- | --- |
| *Total costs ($)* | *24,460,665,837* | *14,771,032,827* | *(9,689,633,010)* |
| *QALYs*  *lost (3% annual disc. rate)* | *4,304,472* | *2,786,419* | *(1,518,053)* |
| *Total COVID-19 cases* | *108,790,250* | *59,015,056* | *(49,775,194)* |
| *Hospitalization days on general ward* | *3,214,401* | *1,768,042* | *(1,446,359)* |
| *Hospitalization days on ICU* | *6,107,399* | *3,616,540* | *(2,490,859)* |
| *Total deaths* | *385,521* | *219,960* | *(165,561)* |
| *Years of life lost (3% annual disc. rate)* | *5,072,737* | *3,265,067* | *(1,807,670)* |
| *Years of life lost (without disc. rate)* | *7,193,633* | *4,874,340* | *(2,319,293)* |
| *QALY lost (without disc. rate)* | *6,210,449* | *4,240,688* | *(1,969,761)* |
| *Total applied vaccines* | *0* | *124,932,625* | *124,932,625* |
| *Vaccination campaign costs* | *-* | *794,571,497* | *794,571,497* |
| *Total COVID-19 event costs* | *24,460,665,837* | *13,976,461,330* | *(10,484,204,507)* |
| *Costs of non-hospitalized symptomatic cases* | *9,014,088,128* | *4,889,840,030* | *(4,124,248,098)* |
| *Hospitalization on general ward costs* | *1,428,769,108* | *785,877,149* | *(642,891,959)* |
| *Hospitalization on ICU costs (without requirement of mechanical ventilation)* | *3,748,736,367* | *2,219,840,658* | *(1,528,895,709)* |
| *Hospitalization on ICU costs (with mechanical ventilation)* | *10,269,072,234* | *6,080,903,493* | *(4,188,168,741)* |

***Table 26. Disaggregated results for Perú - Standard Vaccination Campaign results***

| ***Outcome*** | ***No Vaccination*** | ***Standard Vaccination Campaign*** | ***Difference*** |
| --- | --- | --- | --- |
| *Total costs ($)* | *3,290,563,102* | *2,630,433,417* | *(660,129,685)* |
| *QALYs*  *lost (3% annual disc. rate)* | *2,121,919* | *1,482,872* | *(639,047)* |
| *Total COVID-19 cases* | *23,566,755* | *17,254,407* | *(6,312,348)* |
| *Hospitalization days on general ward* | *774,476* | *517,098* | *(257,378)* |
| *Hospitalization days on ICU* | *1,532,400* | *1,005,169* | *(527,231)* |
| *Total deaths* | *183,735* | *112,688* | *(71,047)* |
| *Years of life lost (3% annual disc. rate)* | *2,543,578* | *1,775,427* | *(768,151)* |
| *Years of life lost (without disc. rate)* | *3,745,417* | *2,740,971* | *(1,004,446)* |
| *QALY lost (without disc. rate)* | *3,118,640* | *2,282,145* | *(836,495)* |
| *Total applied vaccines* | *0* | *31,234,864* | *31,234,864* |
| *Vaccination campaign costs* | *-* | *276,740,892* | *276,740,892* |
| *Total COVID-19 event costs* | *3,290,563,102* | *2,353,692,525* | *(936,870,577)* |
| *Costs of non-hospitalized symptomatic cases* | *2,535,570,724* | *1,856,418,880* | *(679,151,844)* |
| *Hospitalization on general ward costs* | *173,970,633* | *116,155,786* | *(57,814,847)* |
| *Hospitalization on ICU costs (without requirement of mechanical ventilation)* | *162,977,839* | *106,904,373* | *(56,073,466)* |
| *Hospitalization on ICU costs (with mechanical ventilation)* | *418,043,906* | *274,213,486* | *(143,830,420)* |

***Table 27. Disaggregated results for Perú - Optimized Vaccination Campaign results***

| ***Outcome*** | ***No Vaccination*** | ***Optimized Vaccination Campaign*** | ***Difference*** |
| --- | --- | --- | --- |
| *Total costs ($)* | *3,290,563,102* | *2,551,224,598* | *(739,338,504)* |
| *QALYs*  *lost (3% annual disc. rate)* | *2,121,919* | *1,243,159* | *(878,760)* |
| *Total COVID-19 cases* | *23,566,755* | *16,225,623* | *(7,341,132)* |
| *Hospitalization days on general ward* | *774,476* | *422,494* | *(351,982)* |
| *Hospitalization days on ICU* | *1,532,400* | *779,300* | *(753,100)* |
| *Total deaths* | *183,735* | *85,056* | *(98,679)* |
| *Years of life lost (3% annual disc. rate)* | *2,543,578* | *1,486,855* | *(1,056,723)* |
| *Years of life lost (without disc. rate)* | *3,745,417* | *2,368,166* | *(1,377,251)* |
| *QALY lost (without disc. rate)* | *3,118,640* | *1,972,016* | *(1,146,624)* |
| *Total applied vaccines* | *0* | *46,852,295* | *46,852,295* |
| *Vaccination campaign costs* | *-* | *415,111,338* | *415,111,338* |
| *Total COVID-19 event costs* | *3,290,563,102* | *2,136,113,261* | *(1,154,449,841)* |
| *Costs of non-hospitalized symptomatic cases* | *2,535,570,724* | *1,745,730,968* | *(789,839,756)* |
| *Hospitalization on general ward costs* | *173,970,633* | *94,904,725* | *(79,065,908)* |
| *Hospitalization on ICU costs (without requirement of mechanical ventilation)* | *162,977,839* | *82,882,088* | *(80,095,751)* |
| *Hospitalization on ICU costs (with mechanical ventilation)* | *418,043,906* | *212,595,479* | *(205,448,427)* |

***Table 28. Disaggregated results for Perú - Realistic Vaccination Campaign results***

| ***Outcome*** | ***No Vaccination*** | ***Realistic Vaccination Campaign*** | ***Difference*** |
| --- | --- | --- | --- |
| *Total costs ($)* | *3,290,563,102* | *2,874,710,013* | *(415,853,089)* |
| *QALYs*  *lost (3% annual disc. rate)* | *2,121,919* | *1,415,122* | *(706,797)* |
| *Total COVID-19 cases* | *23,566,755* | *16,956,600* | *(6,610,155)* |
| *Hospitalization days on general ward* | *774,476* | *490,061* | *(284,415)* |
| *Hospitalization days on ICU* | *1,532,400* | *938,648* | *(593,752)* |
| *Total deaths* | *183,735* | *104,944* | *(78,791)* |
| *Years of life lost (3% annual disc. rate)* | *2,543,578* | *1,693,923* | *(849,655)* |
| *Years of life lost (without disc. rate)* | *3,745,417* | *2,635,563* | *(1,109,854)* |
| *QALY lost (without disc. rate)* | *3,118,640* | *2,194,402* | *(924,238)* |
| *Total applied vaccines* | *0* | *35,180,868* | *35,180,868* |
| *Vaccination campaign costs* | *-* | *584,354,219* | *584,354,219* |
| *Total COVID-19 event costs* | *3,290,563,102* | *2,290,355,794* | *(1,000,207,308)* |
| *Costs of non-hospitalized symptomatic cases* | *2,535,570,724* | *1,824,377,518* | *(711,193,206)* |
| *Hospitalization on general ward costs* | *173,970,633* | *110,082,325* | *(63,888,308)* |
| *Hospitalization on ICU costs (without requirement of mechanical ventilation)* | *162,977,839* | *99,829,574* | *(63,148,265)* |
| *Hospitalization on ICU costs (with mechanical ventilation)* | *418,043,906* | *256,066,377* | *(161,977,529)* |

**Deterministic sensitivity analysis**

Variables under analysis:

- Vaccine efficacy: +/- 12% of the base case value.

- Vaccination cost per dose:  +/- 25% of the base case value.

- Vaccine coverage: +/- 25% of the base case value.

- Health event costs: +/- 25% of the base case value.

- Disease transmission: +/- 25% of the base case value.

- Vaccine immunity duration: range from 270 to 365 days.

**Table 29. Deterministic sensitivity analysis for Argentina case. Costs are expressed in American dollars for November 2021.**

|  | **No Vaccination** | **Standard Vaccination Campaign** | | **Base case  (Realistic Vaccination Campaign)** | | **Optimized Vaccination Campaign** | |
| --- | --- | --- | --- | --- | --- | --- | --- |
|  |  | **Outcomes** | **ICER** | **Outcomes** | **ICER** | **Outcomes** | **ICER** |
| ***Main results (QALYs lost and Total Costs)*** | | | | | | | |
| **Vaccine Efficacy (Range -/+ 12%)** | | | | | | | |
| **QALYs lost*** | **1,678,317**  **to 1,678,317** | **1,103,179**  **to 1,094,774** | **Cost-saving** | **942,963**  **to 938,798** | **Cost-saving** | **868,370**  **to 867,678** | **Cost-saving** |
| **Total costs ($)** | **$2,489,103,233**  **to $2,489,103,233** | **$2,256,035,130**  **to $2,233,696,614** |  | **$2,231,050,504**  **to $2,213,776,474** |  | **$2,161,134,467**  **to $2,158,043,410** |  |
| **Vaccination cost per dose, including total vaccination costs (Range -/+ 25%)** | | | | | | | |
| **QALYs lost*** | **1,678,317**  **to 1,678,317** | **1,098,725**  **to 1,098,725** | **Cost-saving** | **940,879**  **to 940,879** | **Cost-saving** | **868024**  **to 868024** | **Cost-saving** |
| **Total costs ($)** | **$2,489,103,233**  **to $2,489,103,233** | **$2,147,733,719**  **to $2,341,507,257** |  | **$2,090,107,124**  **to $2,354,589,778** |  | **$2,014,258,822**  **to $2,304,919,129** |  |
| **Vaccination coverage (Range -/+ 25%)** | | | | | | | |
| **QALYs lost*** | **1,678,317**  **to 1,678,317** | **1,221,492**  **to 987,281** | **Cost-saving** | **1,088,082**  **to 811,750** | **Cost-saving** | **1,019,391**  **to 739,604** | **Cost-saving** |
| **Total costs ($)** | **$2,489,103,233**  **to $2,489,103,233** | **$2,264,423,252**  **to $2,236,750,550** |  | **$2,231,305,913**  **to $2,230,517,301** |  | **$2,166,969,196**  **to $2,174,759,500** |  |
| **Health event costs (Range -/+ 25%)** | | | | | | | |
| **QALYs lost*** | **1,678,317**  **to 1,678,317** | **1,098,725**  **to 1,098,725** | **Cost-saving** | **940,879**  **to 940,879** | **Cost-saving** | **868024**  **to 868024** | **Cost-saving** |
| **Total costs ($)** | **$1,866,827,425**  **to $3,111,379,041** | **$1,780,352,135**  **to $2,708,888,841** |  | **$1,799,002,665**  **to $2,645,694,237** |  | **$1,765,021,885**  **to $2,554,156,066** |  |
| **Disease transmission (Range -/+ 25%)** | | | | | | | |
| **QALYs lost*** | **1,003,799**  **to 2,160,870** | **505,093**  **to 1,524,614** | **Cost-saving** | **398,100**  **to 1,356,418** | **Cost-saving** | **342865**  **to 1285107** | **Cost-saving** |
| **Total costs ($)** | **$1,516,594,970**  **to $3,805,171,195** | **$1,276,849,555**  **to $2,846,926,181** |  | **$1,275,485,156**  **to $2,819,179,692** |  | **$1,223,574,946**  **to $2,769,003,122** |  |
| **Vaccine Immunity Duration (Range 270 to 365 days)** | | | | | | | |
| **QALYs lost*** | **1,678,317**  **to 1,678,317** | **1,114,165**  **to 1,089,229** | **Cost-saving** | **958,374**  **to 930,169** | **Cost-saving** | **885084**  **to 857634** | **Cost-saving** |
| **Total costs ($)** | **$2,489,103,233**  **to $2,489,103,233** | **$2,259,226,699**  **to $2,235,745,414** |  | **$2,238,875,243**  **to $2,212,303,055** |  | **$2,176,338,473**  **to $2,149,389,332** |  |

**Table 30. Deterministic sensitivity analysis for Chile case. Costs are expressed in American dollars for November 2021.**

|  | **No Vaccination** | **Standard Vaccination Campaign** | | **Base case  (Realistic Vaccination Campaign)** | | **Optimized Vaccination Campaign** | |
| --- | --- | --- | --- | --- | --- | --- | --- |
|  |  | **Outcomes** | **ICER** | **Outcomes** | **ICER** | **Outcomes** | **ICER** |
| ***Main results (QALYs lost and Total Costs)*** | | | | | | | |
| **Vaccine Efficacy (Range -/+ 12%)** | | | | | | | |
| **QALYs lost*** | **423,399**  **to 423,399** | **315,945**  **to 314,629** | **Cost-saving** | **280,560**  **to 278,071** | **$69 per QALY gained**  **to cost-saving** | **272,707**  **to 272,628** | **Cost-saving** |
| **Total costs ($)** | **1,426,693,488**  **to 1,426,693,488** | **1,350,652,562**  **to 1,342,557,743** |  | **1,436,568,609**  **to 1,423,482,993** |  | **1,325,014,433**  **to 1,323,794,190** |  |
| **Vaccination cost per dose, including total vaccination costs (Range -/+ 25%)** | | | | | | | |
| **QALYs lost*** | **423,399**  **to 423,399** | **315,238**  **to 315,238** | **Cost-saving** | **279,142**  **to 279,142** | **Cost-saving to**  **$538 per QALY gained** | **272,668**  **to 272,668** | **Cost-saving** |
| **Total costs ($)** | **1,426,693,488**  **to 1,426,693,488** | **1,307,272,390**  **to 1,385,816,241** |  | **1,355,486,935**  **to 1,504,235,779** |  | **1,265,496,279**  **to 1,383,312,054** |  |
| **Vaccination coverage (Range -/+ 25%)** | | | | | | | |
| **QALYs lost*** | **423,399**  **to 423,399** | **338,242**  **to 294,191** | **Cost-saving** | **308,356**  **to 253,096** | **Cost-saving to**  **$192 per QALY gained** | **301,535**  **to 247,564** | **Cost-saving** |
| **Total costs ($)** | **1,426,693,488**  **to 1,426,693,488** | **1,349,300,414**  **to 1,348,192,436** |  | **1,406,108,218**  **to 1,459,339,131** |  | **1,321,109,647**  **to 1,334,938,731** |  |
| **Health event costs (Range -/+ 25%)** | | | | | | | |
| **QALYs lost*** | **423,399**  **to 423,399** | **315,238**  **to 315,238** | **Cost-saving** | **279,142**  **to 279,142** | **$532 per QALY gained**  **to cost-saving** | **272,668**  **to 272,668** | **Cost-saving** |
| **Total costs ($)** | **1,070,020,116**  **to 1,783,366,860** | **1,049,180,162**  **to 1,643,908,469** |  | **1,146,770,439**  **to 1,712,952,274** |  | **1,052,211,013**  **to 1,596,597,320** |  |
| **Disease transmission (Range -/+ 25%)** | | | | | | | |
| **QALYs lost*** | **267,530**  **to 528,997** | **169,871**  **to 417,106** | **Cost-saving** | **143,272**  **to 379,220** | **Cost-saving** | **136,577**  **to 373,255** | **Cost-saving** |
| **Total costs ($)** | **926,073,471**  **to 1,957,830,642** | **831,048,995**  **to 1,658,832,068** |  | **913,120,886**  **to 1,742,133,347** |  | **803,968,334**  **to 1,640,194,651** |  |
| **Vaccine Immunity Duration (Range 270 to 365 days)** | | | | | | | |
| **QALYs lost*** | **423,399**  **to 423,399** | **317,539**  **to 313,817** | **Cost-saving** | **281,907**  **to 277,444** | **$55 per QALY gained**  **to $2 per QALY gained** | **275,304**  **to 271,059** | **Cost-saving** |
| **Total costs ($)** | **1,426,693,488**  **to 1,426,693,488** | **1,350,695,689**  **to 1,343,990,997** |  | **1,434,454,549**  **to 1,427,046,056** |  | **1,329,336,704**  **to 1,321,396,088** |  |

**Table 31. Deterministic sensitivity analysis for Colombia case. Costs are expressed in American dollars for November 2021.**

|  | **No Vaccination** | **Standard Vaccination Campaign** | | **Base case  (Realistic Vaccination Campaign)** | | **Optimized Vaccination Campaign** | |
| --- | --- | --- | --- | --- | --- | --- | --- |
|  |  | **Outcomes** | **ICER** | **Outcomes** | **ICER** | **Outcomes** | **ICER** |
| ***Main results (QALYs lost and Total Costs)*** | | | | | | | |
| **Vaccine Efficacy (Range -/+ 12%)** | | | | | | | |
| **QALYs lost*** | **1,878,855 to 1,878,855** | **1,340,410 to 1,334,557** | **Cost-saving** | **1,249,170 to 1,244,119** | **Cost-saving** | **1,136,425 to 1,136,110** | **Cost-saving** |
| **Total costs ($)** | **5,213,429,639 to 5,213,429,639** | **4,007,537,676 to 3,979,171,861** |  | **4,284,234,462 to 4,257,557,589** |  | **3,856,684,517 to 3,853,053,884** |  |
| **Vaccination cost per dose, including total vaccination costs (Range -/+ 25%)** | | | | | | | |
| **QALYs lost*** | **1,878,855 to 1878855** | **1,337,234 to 1,337,234** | **Cost-saving** | **1,246,398 to 1,246,398** | **Cost-saving** | **1,136,268 to 1,136,268** | **Cost-saving** |
| **Total costs ($)** | **5,213,429,639 to 5,213,429,639** | **3,883,530,865 to 4,102,423,483** |  | **4,057,875,103 to 4,483,291,053** |  | **3,690,699,836 to 4,019,038,763** |  |
| **Vaccination coverage (Range -/+ 25%)** | | | | | | | |
| **QALYs lost*** | **1,878,855 to 1,878,855** | **1,444,771 to 1,238,000** | **Cost-saving** | **1,369,679 to 1,133,905** | **Cost-saving** | **1,274,117 to 1,013,990** | **Cost-saving** |
| **Total costs ($)** | **5,213,429,639 to 5,213,429,639** | **4,047,489,149 to 3,963,879,115** |  | **4,232,287,817 to 4,329,250,120** |  | **3,892,236,781 to 3,840,860,050** |  |
| **Health event costs (Range -/+ 25%)** | | | | | | | |
| **QALYs lost*** | **1,878,855 to 1,878,855** | **1,337,234 to 1,337,234** | **Cost-saving** | **1,246,398 to 1,246,398** | **Cost-saving** | **1,136,268 to 1,136,268** | **Cost-saving** |
| **Total costs ($)** | **3,910,072,229 to 6,516,787,048** | **3,104,179,190 to 4,881,775,159** |  | **3,415,645,283 to 5,125,520,872** |  | **3,055,321,438 to 4,654,417,160** |  |
| **Disease transmission (Range -/+ 25%)** | | | | | | | |
| **QALYs lost*** | **1,211,609 to 2,473,442** | **758,655 to 1,752,648** | **Cost-saving** | **686,174 to 1,657,400** | **Cost-saving** | **598,217 to 1,543,348** | **Cost-saving** |
| **Total costs ($)** | **3,018,295,725 to 8,416,418,135** | **2,564,372,085 to 4,959,632,148** |  | **2,844,971,762 to 5,191,891,400** |  | **2,439,551,686 to 4,767,007,299** |  |
| **Vaccine Immunity Duration (Range 270 to 365 days)** | | | | | | | |
| **QALYs lost*** | **1,878,855 to 1,878,855** | **1,347,812 to 1,330,730** | **Cost-saving** | **1,258,046 to 1,239,219** | **Cost-saving** | **1,148,668 to 1,128,683** | **Cost-saving** |
| **Total costs ($)** | **5,213,429,639 to 5,213,429,639** | **4,009,866,693 to 3,982,902,329** |  | **4,286,963,013 to 4,260,501,802** |  | **3,872,895,726 to 3,843,853,698** |  |

**Table 32. Deterministic sensitivity analysis for Costa Rica case. Costs are expressed in American dollars for November 2021.**

|  | **No Vaccination** | **Standard Vaccination Campaign** | | **Base case  (Realistic Vaccination Campaign)** | | **Optimized Vaccination Campaign** | |
| --- | --- | --- | --- | --- | --- | --- | --- |
|  |  | **Outcomes** | **ICER** | **Outcomes** | **ICER** | **Outcomes** | **ICER** |
| ***Main results (QALYs lost and Total Costs)*** | | | | | | | |
| **Vaccine Efficacy (Range -/+ 12%)** | | | | | | | |
| **QALYs lost*** | **130,323 to 130,323** | **89,368 to 88,907** | **Cost-saving** | **80,778 to 80,626** | **Cost-saving** | **73,895 to 73,870** | **Cost-saving** |
| **Total costs ($)** | **414,660,479 to 414,660,479** | **314,609,194 to 311,999,570** |  | **320,041,450 to 318,754,315** |  | **302,667,288 to 302,331,485** |  |
| **Vaccination cost per dose, including total vaccination costs (Range -/+ 25%)** | | | | | | | |
| **QALYs lost*** | **130,323 to 130,323** | **89,120 to 89,120** | **Cost-saving** | **80,698 to 80,698** | **Cost-saving** | **73,883 to 73,883** | **Cost-saving** |
| **Total costs ($)** | **414,660,479 to 414,660,479** | **302,705,825 to 323,854,342** |  | **302,914,546 to 335,864,937** |  | **286,638,024 to 318,360,800** |  |
| **Vaccination coverage (Range -/+ 25%)** | | | | | | | |
| **QALYs lost*** | **130,323 to 130,323** | **97,298 to 81,601** | **Cost-saving** | **90,170 to 72,170** | **Cost-saving** | **84,265 to 64,749** | **Cost-saving** |
| **Total costs ($)** | **414,660,479 to 414,660,479** | **317,315,092 to 311,558,573** |  | **318,836,571 to 321,745,330** |  | **304,447,129 to 302,736,501** |  |
| **Health event costs (Range -/+ 25%)** | | | | | | | |
| **QALYs lost*** | **130,323 to 130,323** | **89,120 to 89,120** | **Cost-saving** | **80,698 to 80,698** | **Cost-saving** | **73,883 to 73,883** | **Cost-saving** |
| **Total costs ($)** | **310,995,359 to 518,325,599** | **245,534,321 to 381,025,846** |  | **256,017,502 to 382,761,981** |  | **242,735,947 to 362,262,877** |  |
| **Disease transmission (Range -/+ 25%)** | | | | | | | |
| **QALYs lost*** | **84,621 to 170,910** | **50,959 to 116,787** | **Cost-saving** | **44,070 to 108,002** | **Cost-saving** | **38,938 to 10,079** | **Cost-saving** |
| **Total costs ($)** | **237,584,694 to 660,620,391** | **204,834,276 to 388,854,354** |  | **211,292,365 to 390,937,722** |  | **196,085,932 to 373,723,371** |  |
| **Vaccine Immunity Duration (Range 270 to 365 days)** | | | | | | | |
| **QALYs lost*** | **130,323 to 130,323** | **89,964 to 88,600** | **Cost-saving** | **81,626 to 80,131** | **Cost-saving** | **74,859 to 73,286** | **Cost-saving** |
| **Total costs ($)** | **414,660,479 to 414,660,479** | **314,824,019 to 312,345,724** |  | **320,925,347 to 318,461,461** |  | **304,141,942 to 301,496,934** |  |

**Table 33. Deterministic sensitivity analysis for México case. Costs are expressed in American dollars for November 2021.**

|  | **No Vaccination** | **Standard Vaccination Campaign** | | **Base case  (Realistic Vaccination Campaign)** | | **Optimized Vaccination Campaign** | |
| --- | --- | --- | --- | --- | --- | --- | --- |
|  |  | **Outcomes** | **ICER** | **Outcomes** | **ICER** | **Outcomes** | **ICER** |
| ***Main results (QALYs lost and Total Costs)*** | | | | | | | |
| **Vaccine Efficacy (Range -/+ 12%)** | | | | | | | |
| **QALYs lost*** | **4,304,472**  **to 4,304,472** | **2,791,631**  **to 2,779,047** | **Cost-saving** | **2,790,160**  **to 2,783,063** | **Cost-saving** | **2,332,632**  **to 2,332,357** | **Cost-saving** |
| **Total costs ($)** | **24,460,665,837**  **to 24,460,665,837** | **15,256,949,199**  **to 15,090,030,079** |  | **14,830,399,884**  **to 14,716,025,495** |  | **13,340,994,703**  **to 13,332,619,586** |  |
| **Vaccination cost per dose, including total vaccination costs (Range -/+ 25%)** | | | | | | | |
| **QALYs lost*** | **4,304,472**  **to 4,304,472** | **2,784,670**  **to 2,784,670** | **Cost-saving** | **2,786,419**  **to 2,786,419** | **Cost-saving** | **2,332,495**  **to 2,332,495** | **Cost-saving** |
| **Total costs ($)** | **24,460,665,837**  **to 24,460,665,837** | **14,889,337,068**  **to 15,448,379,018** |  | **14,572,389,953**  **to 14,969,675,702** |  | **12,917,525,940**  **to 13,756,088,865** |  |
| **Vaccination coverage (Range -/+ 25%)** | | | | | | | |
| **QALYs lost*** | **4,304,472**  **to 4,304,472** | **3,038,215**  **to 2,556,117** | **Cost-saving** | **3,038,488**  **to 2,559,290** | **Cost-saving** | **2,644,766**  **to 2,057,405** | **Cost-saving** |
| **Total costs ($)** | **24,460,665,837**  **to 24,460,665,837** | **16,126,579,253**  **to 14,414,502,437** |  | **15,812,413,098**  **to 13,928,082,119** |  | **14,364,028,394**  **to 12,480,870,358** |  |
| **Health event costs (Range -/+ 25%)** | | | | | | | |
| **QALYs lost*** | **4,304,472**  **to 4,304,472** | **2,784,670**  **to 2,784,670** | **Cost-saving** | **2,786,419**  **to 2,786,419** | **Cost-saving** | **2,332,495**  **to 2,332,495** | **Cost-saving** |
| **Total costs ($)** | **18,345,499,378**  **to 30,575,832,297** | **11,656,164,507**  **to 18,681,551,579** |  | **11,276,917,495**  **to 18,265,148,160** |  | **10,421,887,014**  **to 16,251,727,791** |  |
| **Disease transmission (Range -/+ 25%)** | | | | | | | |
| **QALYs lost*** | **2,611,916**  **to 5,768,799** | **1,569,382**  **to 3,698,314** | **Cost-saving** | **1,569,402**  **to 3,700,715** | **Cost-saving** | **1,263,854**  **to 3,197,154** | **Cost-saving** |
| **Total costs ($)** | **13,118,327,865**  **to 36,031,796,302** | **8,979,888,934**  **to 19,954,413,582** |  | **8,614,527,725**  **to 19,509,809,821** |  | **7,938,692,009**  **to 17,527,767,774** |  |
| **Vaccine Immunity Duration (Range 270 to 365 days)** | | | | | | | |
| **QALYs lost*** | **4,304,472**  **to 4,304,472** | **2,810,523**  **to 2,769,077** | **Cost-saving** | **2,812,063**  **to 2,770,914** | **Cost-saving** | **2,360,296**  **to 2,315,527** | **Cost-saving** |
| **Total costs ($)** | **24,460,665,837**  **to 24,460,665,837** | **15,307,211,059**  **to 15,088,220,392** |  | **14,909,165,035**  **to 14,689,908,747** |  | **13,474,188,861**  **to 13,252,879,267** |  |

**Table 34. Deterministic sensitivity analysis for the Perú case. Costs are expressed in American dollars for November 2021.**

|  | **No Vaccination** | **Standard Vaccination Campaign** | | **Base case  (Realistic Vaccination Campaign)** | | **Optimized Vaccination Campaign** | |
| --- | --- | --- | --- | --- | --- | --- | --- |
|  |  | **Outcomes** | **ICER** | **Outcomes** | **ICER** | **Outcomes** | **ICER** |
| ***Main results (QALYs lost and Total Costs)*** | | | | | | | |
| **Vaccine Efficacy (Range -/+ 12%)** | | | | | | | |
| **QALYs lost*** | **2,121,919**  **to 2,121,919** | **1,486,144**  **to 1,480,285** | **Cost-saving** | **1,416,214**  **to 1,414,182** | **Cost-saving** | **1,243,172**  **to 1,243,146** | **Cost-saving** |
| **Total costs ($)** | **3,290,563,102**  **to 3,290,563,102** | **2,639,409,280**  **to 2,621,892,949** |  | **2,880,488,697**  **to 2,869,001,340** |  | **2,552,417,128**  **to 2,550,031,780** |  |
| **Vaccination cost per dose, including total vaccination costs (Range -/+ 25%)** | | | | | | | |
| **QALYs lost*** | **2,121,919**  **to 2,121,919** | **1,482,872**  **to 1,482,872** | **Cost-saving** | **1,415,122**  **to 1,415,122** | **Cost-saving** | **124,315**  **to 1,243,159** | **Cost-saving** |
| **Total costs ($)** | **3,290,563,102**  **to 3,290,563,102** | **2,561,248,194**  **to 2,699,618,640** |  | **2,728,621,458**  **to 3,020,798,567** |  | **2,447,446,764**  **to 2,655,002,433** |  |
| **Vaccination coverage (Range -/+ 25%)** | | | | | | | |
| **QALYs lost*** | **2,121,919**  **to 2,121,919** | **1,614,338**  **to 1,361,047** | **Cost-saving** | **1,557,744**  **to 1,284,247** | **Cost-saving** | **1,411,385**  **to 1,093,576** | **Cost-saving** |
| **Total costs ($)** | **3,290,563,102**  **to 3,290,563,102** | **2,658,010,099**  **to 2,616,467,297** |  | **2,829,901,725**  **to 2,930,705,972** |  | **2,569,769,210**  **to 2,546,547,893** |  |
| **Health event costs (Range -/+ 25%)** | | | | | | | |
| **QALYs lost*** | **2,121,919**  **to 2,121,919** | **1,482,872**  **to 1,482,872** | **Cost-saving** | **1,415,122**  **to 1,415,122** | **Cost-saving** | **1,243,159**  **to 1,243,159** | **Cost-saving** |
| **Total costs ($)** | **2,467,922,326**  **to 4,113,203,877** | **2,042,010,285**  **to 3,218,856,548** |  | **2,302,121,064**  **to 3,447,298,961** |  | **2,017,196,283**  **to 3,085,252,913** |  |
| **Disease transmission (Range -/+ 25%)** | | | | | | | |
| **QALYs lost*** | **1,412,225**  **to 2,686,552** | **871,861**  **to 1,918,567** | **Cost-saving** | **817,015**  **to 1,849,181** | **Cost-saving** | **689,347**  **to 1,670,655** | **Cost-saving** |
| **Total costs ($)** | **2,004,532,543**  **to 5,313,364,357** | **1,714,673,026**  **to 3,212,908,949** |  | **1,955,575,811**  **to 3,445,206,607** |  | **1,642,919,431**  **to 3,121,835,801** |  |
| **Vaccine Immunity Duration (Range 270 to 365 days)** | | | | | | | |
| **QALYs lost*** | **2,121,919**  **to 2,121,919** | **1,494,728**  **to 1,475,626** | **Cost-saving** | **1,427,590**  **to 1,407,464** | **Cost-saving** | **1,257,051**  **to 1,234,671** | **Cost-saving** |
| **Total costs ($)** | **3,290,563,102**  **to 3,290,563,102** | **2,639,587,427**  **to 2,625,076,117** |  | **2,883,502,649**  **to 2,869,284,320** |  | **2,561,155,434**  **to 2,545,163,344** |  |

Model calibration

Calibration of the SEIR model involved comparing the number of deaths reported by official sites for each country and each age group. Particularly, we applied different multiplicative factors to the case base infectious fatality rate (ifr), for each of the countries:

Argentina = base ifr value of each age group * 2.4

Colombia = base ifr value of each age group * 1.8

Mexico = base ifr value of each age group * 1.8

Perú = base ifr value of each age group * 3.55

It was not necessary to adjust for a multiplicative factor for Brazil, Chile, and Costa Rica.

Interactive online CEA model

As already mentioned, the model is freely available at the following link: ​​<https://iecs.shinyapps.io/seir_ages_CEA_paper/>.

Given that most of the countries involved in the model use Spanish as their official language, it has been mainly developed in this language. Figure 1 presents a screenshot of the interactive web model. In particular, we show the interactive deterministic sensitivity analysis for the economic evaluation presented in this article. This shiny application allows the user to see the results of QALYs lost and total costs of each scenario, for the corresponding country, after having applied different degrees of uncertainty to any of the variables taken into account in the sensitivity analysis.

Figure 1. Example screenshot of the online interactive deterministic sensitivity analysis.


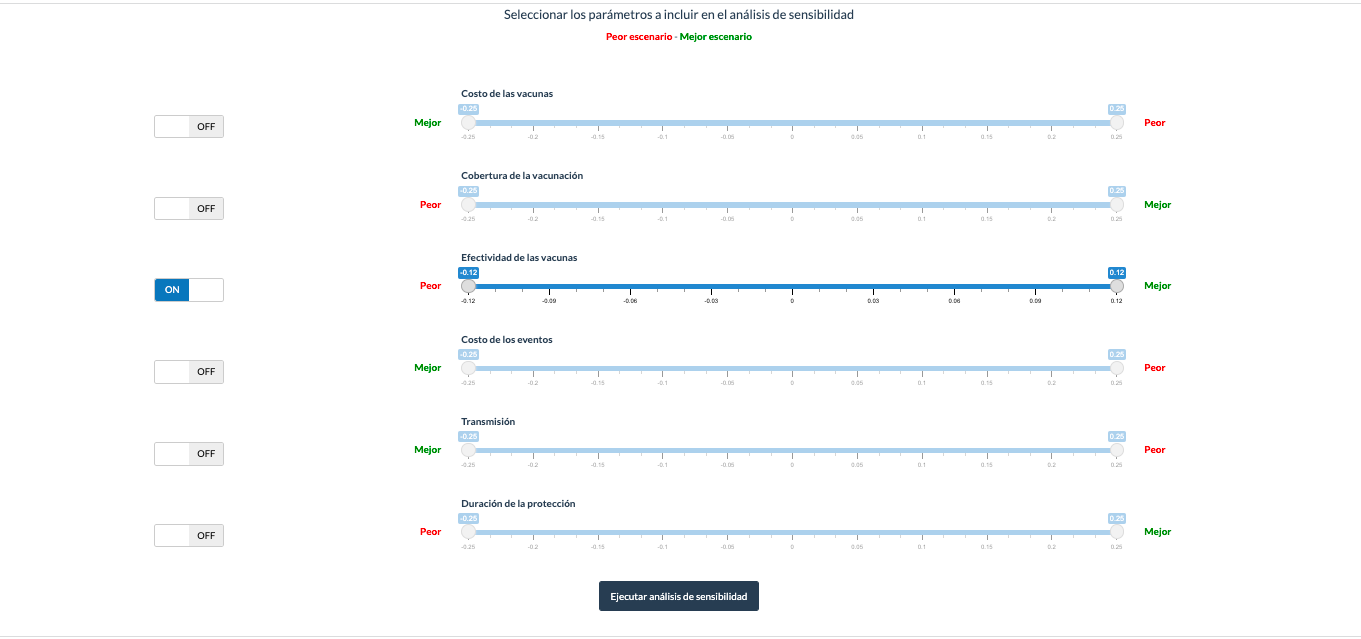


ADVISORY BOARD

​​**Project: RG-E1696**

**“Integrated Healthcare Preparedness Model to estimate the impact of the expansion of COVID-19”.**

**Michael Reich:** Politólogo estadounidense y profesor del Programa Taro Takemi sobre International Health Policy en la [escuela de salud pública Harvard TH Chan](https://en.wikipedia.org/wiki/Harvard_T.H._Chan_School_of_Public_Health) en Boston, Massachusetts. Especialista en análisis político de la reforma de salud, política farmacéutica y acceso a medicamentos así como en política de salud de Japón y México. Ha sido director del Curso emblemático del Banco Mundial sobre la reforma del sector de la salud y el financiamiento sostenible (Flagship Course on Health Sector Reform and Sustainable Financing) Como parte de esta colaboración, fue coautor de un libro histórico sobre sistemas de salud: Cómo lograr una reforma de salud correcta: una guía para mejorar el rendimiento y la equidad. En 2016, Michael Reich recibió el Premio Award for Lifetime Service to the Field of Health Policy and Systems Research otorgado por Alliance for Health Policy and Systems Research and from Health Systems Global.

**Emilio Santelices:** médico y académico chileno. Doctorado en [salud pública](https://es.wikipedia.org/wiki/Salud_p%C3%BAblica) y diplomado en administración de instituciones de salud en la [Universidad de Chile](https://es.wikipedia.org/wiki/Universidad_de_Chile). [MBA](https://es.wikipedia.org/wiki/Maestr%C3%ADa_en_Administraci%C3%B3n_de_Empresas) de la [Universidad Tulane](https://es.wikipedia.org/wiki/Universidad_Tulane), [Estados Unidos](https://es.wikipedia.org/wiki/Estados_Unidos). Entre los años 2010 y 2014 fue asesor de gabinete del Ministerio de Salud de Chile en temas de mejoras y evaluación de calidad para hospitales y centros de salud primaria. En 2018 y 2019 fue designado ministro de Salud por el presidente Sebastián Piñera^.^

**Alarico Rodriguez:** Médico. Fue Gerente General de la Administración de Servicios de Salud del Estado (ASSE) de Uruguay (2018 2020); Director Técnico Fondo Nacional de Recursos (FNR) de Uruguay (2011 - 2018), previamente Sub-Director Técnico de Prestaciones (1998 – 2011); Director 1727 Emergencias (1998 – 2003). Se desempeñó como Asesor en Definición de Servicios de Salud para OPS/OMS, Washington (2015 – 2016); Coordinador Académico del curso Diseño y Gestión de Conjuntos de Prestaciones de Salud en Campus Virtual de OPS / OMS (2012 -2015); Asesor del Gobierno de Guyana en la actualización del "Package of Publically Guaranteed Health Services" (2012 – 2013); Consultor del Ministerio de Salud de Ecuador (2012 – 2013); Representante de la Región Latinoamérica en el Consejo de la Asociación Médica Mundial (2003 – 2018); Miembro del Consejo Asesor Editorial de Value in Health Regional Issues (2015 – 2017); Miembro del Comité Ejecutivo de la Red de Evaluación de Tecnologías Sanitarias de las Américas – REDETSA (2015 – 2017); entre otros cargos.

**Fernando Pio De La Hoz Restrepo**: Médico de la Universidad Libre De Colombia Barranquilla, magíster en epidemiología de la Universidad del Valle y doctor en epidemiología de enfermedades infecciosas de la Universidad de Londres y epidemiólogo de campo del Programa de Epidemiología Aplicada del Instituto Nacional de Salud. Fue Director General del Instituto Nacional de Salud, trabajó en el Departamento Administrativo de Ciencia, Tecnología e Innovación (Colciencias) fue profesor e investigador principal en la Universidad Nacional de Colombia, en Escuela de Medicina Juan N. Corpas y en el Instituto Nacional de Salud. Fue coordinador del Centro Control de Enfermedades, Subdirector de Epidemiología y Laboratorio Nacional de Referencia e Investigador Científico, entre otras.

**Jeremy Veillard:** Especialista Senior en Salud en la región de América Latina y el Caribe de la Práctica Global de Salud, Nutrición y Población del Banco Mundial. Es un ex ejecutivo de atención médica en Canadá y Francia, con amplia experiencia en calidad de atención, prestación de servicios e investigación y análisis de sistemas de salud. Doctorado en salud pública (investigación de sistemas de salud) de la Universidad de Amsterdam, Países Bajos y magister en administración de hospitales de la Ecole des Hautes Etudes en Santé Publique, Francia.

**Patricia García Funegra:** Médica de la Universidad Peruana Cayetano Heredia, Magister en Salud Pública por la Universidad de Washington y Doctora en Medicina por la Universidad Peruana Cayetano Heredia. A lo largo de su carrera se ha especializado en salud reproductiva y enfermedades de transmisión sexual. Ha trabajado en epidemiología, salud pública e investigación sobre la implementación y mejora de la calidad de los servicios. fue miembro del Comité Consultivo de Expertos de Salud Reproductiva y Presidenta del Comité de Expertos Internacionales para vacunas contra el [virus del papiloma humano](https://es.wikipedia.org/wiki/Virus_del_papiloma_humano) entre el 2007-2008 de la OMS. Durante 2011-2016 se desempeñó como Decana de la Facultad de Salud Pública y Administración (FASPA) de la Universidad Peruana Cayetano Heredia, donde es docente e investigadora. También es Profesora Principal en investigación en Infecciones de Transmisión Sexual, adjunta de Salud Global en la Universidad de Washington y de Epidemiología de la [Universidad Tulane](https://es.wikipedia.org/wiki/Universidad_Tulane). Ha sido jefa del Instituto Nacional de Salud del Perú y también Ministra de Salud entre el año 2016 y 2017.

**Cristina Guimaraes:** Es Licenciada en Economía y Doctora en Demografía y Estudios de la Población por la Universidad Federal de Minas Gerais. Es coordinadora del MBA en Economía y Evaluación de Tecnologías Sanitarias de la Fundación Instituto de Economía de la Universidad de Sao Paulo, donde también es profesora de Salud Pública y Demografía. A lo largo de su carrera se ha desarrollado en investigación en demografía orientada a la salud pública y en consultoría para políticas públicas de salud.

**Felicia Knaul:** Licenciada en Desarrollo Internacional por la Universidad de Toronto y Doctora en Economía por la Universidad de Harvard. Directora del Instituto para Estudios Avanzados de las Américas de la Universidad de Miami y progesora en la Escuela de Medicina Leonard M. Miller. Actualmente es presidenta de la Comisión Lancet sobre Violencia de Género y Maltrato a los Jóvenes. Tiene más de 20 años de experiencia en organizaciones gubernamentales, académicas, de grupos de expertos y de la sociedad civil. Ha ocupado altos cargos en los gobiernos de México y Colombia, y ha trabajado para varias agencias internacionales, incluida la Organización Mundial de la Salud. Hasta agosto de 2015, se desempeñó como profesora asociada en la Escuela de Medicina de Harvard y directora de la Iniciativa de Equidad Global de Harvard. Su investigación se centra en la salud global, el cáncer global y especialmente el cáncer de mama, la participación femenina en la fuerza laboral, la mujer y la salud, los sistemas de salud y sus reformas, la financiación de la salud y el acceso al control del dolor y los cuidados paliativos.

**Marisa Santos:** Médica infectóloga y Doctora en Epidemiología por la Universidad del Estado de Río de Janeiro. Desde 2009 es coordinadora del NATS (Centro de Evaluación de Tecnologías Sanitarias) del Instituto Nacional de Cardiología de Brasil. Es consultora en Epidemiología, Evaluación de Tecnologías Sanitarias con experiencia directa en Modelos de Predicción y Práctica Clínica Basada en Evidencia. Su trabajo se ha enfocado en infecciones, epidemias, creación de guías, comparaciones indirectas, revisiones sistemáticas, costo-utilidad.

**Álvaro Gutiérrez Áviles:** Es magíster en Salud Pública por la Universidad Mayor de San Andrés, Bolivia. Actualmente es consultor en Salud Pública del Banco Interamericano de Desarrollo. A lo largo de su carrera se ha especializado en el gerenciamiento de proyectos con financiamiento internacional. monitoreo y evaluación de proyectos de salud, redes de salud y epidemiología, así como en la gestión de recursos humanos en salud.

**Valentina Vargas:** Es licenciada en Salud Pública de la Universidad de Miami y Magister en Salud Pública y Población por la Universidad de Harvard. Actualmente es promotora de Salud Global en la Escuela de Salud Pública de Harvard y actualmente se desarrolla como Directora de Investigación en el Instituto de Estudios Avanzados de las Américas de la Universidad de Miami.

*References*

1. [González S, Olszevicki S, Salazar M, Calabria A, Regairaz L, Marín L, et al. Effectiveness of the first component of Gam-COVID-Vac (Sputnik V) on reduction of SARS-CoV-2 confirmed infections, hospitalisations and mortality in patients aged 60-79: a retrospective cohort study in Argentina. EClinicalMedicine. 2021;40: 101126. doi:](http://paperpile.com/b/0ylhSY/pURy)[10.1016/j.eclinm.2021.101126](http://dx.doi.org/10.1016/j.eclinm.2021.101126)

2. [Logunov DY, Dolzhikova IV, Shcheblyakov DV, Tukhvatulin AI, Zubkova OV, Dzharullaeva AS, et al. Safety and efficacy of an rAd26 and rAd5 vector-based heterologous prime-boost COVID-19 vaccine: an interim analysis of a randomised controlled phase 3 trial in Russia. Lancet. 2021;397: 671–681. doi:](http://paperpile.com/b/0ylhSY/HBZ6)[10.1016/S0140-6736(21)00234-8](http://dx.doi.org/10.1016/S0140-6736(21)00234-8)

3. [Agency for Clinical Innovation. Living evidence - COVID-19 vaccines. In: Agency for Clinical Innovation [Internet]. 22 Mar 2022 [cited 22 Mar 2022]. Available:](http://paperpile.com/b/0ylhSY/wtKK) <https://aci.health.nsw.gov.au/covid-19/critical-intelligence-unit/covid-19-vaccines>

4. [Al Kaabi N, Zhang Y, Xia S, Yang Y, Al Qahtani MM, Abdulrazzaq N, et al. Effect of 2 Inactivated SARS-CoV-2 Vaccines on Symptomatic COVID-19 Infection in Adults: A Randomized Clinical Trial. JAMA. 2021;326: 35–45. doi:](http://paperpile.com/b/0ylhSY/HwyU)[10.1001/jama.2021.8565](http://dx.doi.org/10.1001/jama.2021.8565)

5. [Palacios R, Batista AP, Albuquerque CSN, Patiño EG, Santos J do P, Tilli Reis Pessoa Conde M, et al. Efficacy and Safety of a COVID-19 Inactivated Vaccine in Healthcare Professionals in Brazil: The PROFISCOV Study. 2021. doi:](http://paperpile.com/b/0ylhSY/02Nv)[10.2139/ssrn.3822780](http://dx.doi.org/10.2139/ssrn.3822780)

6. [Jara A, Undurraga EA, González C, Paredes F, Fontecilla T, Jara G, et al. Effectiveness of an Inactivated SARS-CoV-2 Vaccine in Chile. N Engl J Med. 2021;385: 875–884. doi:](http://paperpile.com/b/0ylhSY/wnnD)[10.1056/NEJMoa2107715](http://dx.doi.org/10.1056/NEJMoa2107715)

7. [Polack FP, Thomas SJ, Kitchin N, Absalon J, Gurtman A, Lockhart S, et al. Safety and Efficacy of the BNT162b2 mRNA Covid-19 Vaccine. N Engl J Med. 2020;383: 2603–2615. doi:](http://paperpile.com/b/0ylhSY/vb2z)[10.1056/NEJMoa2034577](http://dx.doi.org/10.1056/NEJMoa2034577)

8. [Haas EJ, Angulo FJ, McLaughlin JM, Anis E, Singer SR, Khan F, et al. Impact and effectiveness of mRNA BNT162b2 vaccine against SARS-CoV-2 infections and COVID-19 cases, hospitalisations, and deaths following a nationwide vaccination campaign in Israel: an observational study using national surveillance data. Lancet. 2021;397: 1819–1829. doi:](http://paperpile.com/b/0ylhSY/2zPV)[10.1016/S0140-6736(21)00947-8](http://dx.doi.org/10.1016/S0140-6736(21)00947-8)

9. [Voysey M, Costa Clemens SA, Madhi SA, Weckx LY, Folegatti PM, Aley PK, et al. Single-dose administration and the influence of the timing of the booster dose on immunogenicity and efficacy of ChAdOx1 nCoV-19 (AZD1222) vaccine: a pooled analysis of four randomised trials. Lancet. 2021;397: 881–891. doi:](http://paperpile.com/b/0ylhSY/MXWs)[10.1016/S0140-6736(21)00432-3](http://dx.doi.org/10.1016/S0140-6736(21)00432-3)

10. [Baden LR, El Sahly HM, Essink B, Kotloff K, Frey S, Novak R, et al. Efficacy and Safety of the mRNA-1273 SARS-CoV-2 Vaccine. N Engl J Med. 2021;384: 403–416. doi:](http://paperpile.com/b/0ylhSY/DLu0)[10.1056/NEJMoa2035389](http://dx.doi.org/10.1056/NEJMoa2035389)

11. [Baraniuk C. What do we know about China’s covid-19 vaccines? BMJ. 2021;373: n912. doi:](http://paperpile.com/b/0ylhSY/Mn4V)[10.1136/bmj.n912](http://dx.doi.org/10.1136/bmj.n912)

12. [Sadoff J, Gray G, Vandebosch A, Cárdenas V, Shukarev G, Grinsztejn B, et al. Safety and Efficacy of Single-Dose Ad26.COV2.S Vaccine against Covid-19. N Engl J Med. 2021;384: 2187–2201. doi:](http://paperpile.com/b/0ylhSY/aWCR)[10.1056/NEJMoa2101544](http://dx.doi.org/10.1056/NEJMoa2101544)

13. [Argentina superará las 20 millones de vacunas recibidas: llegan hoy 811.000 AstraZeneca desde México y el lunes 934.200 desde EEUU. In: Argentina.gob.ar [Internet]. 12 Jun 2021 [cited 22 Mar 2022]. Available:](http://paperpile.com/b/0ylhSY/0Mbr) <https://www.argentina.gob.ar/noticias/argentina-superara-las-20-millones-de-vacunas-recibidas-llegan-hoy-811000-astrazeneca-desde>

14. [Brenes L. Llegó al país primer lote de vacunas contra COVID-19. In: Ministerio de Salud Costa Rica [Internet]. [cited 22 Mar 2022]. Available:](http://paperpile.com/b/0ylhSY/FkRi) <https://www.ministeriodesalud.go.cr/index.php/centro-de-prensa/noticias/741-noticias-2020/2019-llego-al-pais-primer-lote-de-vacunas-contra-covid-19>

15. [Piñera celebra anuncio de Pfizer y el lugar prioritario de Chile en la fila de espera por su vacuna. Reuters. 9 Nov 2020. Available:](http://paperpile.com/b/0ylhSY/eiLw) <https://www.reuters.com/article/salud-coronavirus-chile-idLTAKBN27P2FU>[. Accessed 22 Mar 2022.](http://paperpile.com/b/0ylhSY/eiLw)

16. [de Colombia M de S y. PS. Minsalud detalló proceso de adquisición de vacunas. [cited 22 Mar 2022]. Available:](http://paperpile.com/b/0ylhSY/9v4C) <https://www.minsalud.gov.co/Paginas/Minsalud-detallo-proceso-de-adquisicion-de-vacunas.aspx>

17. [Vacinação contra a Covid-19 no Brasil - #PÁTRIAVACINADA. [cited 22 Mar 2022]. Available:](http://paperpile.com/b/0ylhSY/QExb) <https://www.gov.br/saude/pt-br/vacinacao>

18. [Gestión diplomática vacunas Covid. [cited 22 Mar 2022]. Available:](http://paperpile.com/b/0ylhSY/zTet) <https://transparencia.sre.gob.mx/gestion-diplomatica-vacunas-covid/>

19. [Gobierno del Perú. [cited 22 Mar 2022]. Available:](http://paperpile.com/b/0ylhSY/YY9o) <https://www.gob.pe/>

20. [Prem K, van Zandvoort K, Klepac P, Eggo RM, Davies NG, Centre for the Mathematical Modelling of Infectious Diseases COVID-19 Working Group, et al. Projecting contact matrices in 177 geographical regions: An update and comparison with empirical data for the COVID-19 era. PLoS Comput Biol. 2021;17: e1009098. doi:](http://paperpile.com/b/0ylhSY/tRTk)[10.1371/journal.pcbi.1009098](http://dx.doi.org/10.1371/journal.pcbi.1009098)

21. [Davies NG, Klepac P, Liu Y, Prem K, Jit M, CMMID COVID-19 working group, et al. Age-dependent effects in the transmission and control of COVID-19 epidemics. Nat Med. 2020;26: 1205–1211. doi:](http://paperpile.com/b/0ylhSY/95fg)[10.1038/s41591-020-0962-9](http://dx.doi.org/10.1038/s41591-020-0962-9)

22. [Lapidus N, Paireau J, Levy-Bruhl D, de Lamballerie X, Severi G, Touvier M, et al. Do not neglect SARS-CoV-2 hospitalization and fatality risks in the middle-aged adult population. Infect Dis Now. 2021;51: 380–382. doi:](http://paperpile.com/b/0ylhSY/CiI6)[10.1016/j.idnow.2020.12.007](http://dx.doi.org/10.1016/j.idnow.2020.12.007)

23. [Szende A, Janssen B, Cabases J. Self-Reported Population Health: An International Perspective based on EQ-5D. Springer; 2013. Available:](http://paperpile.com/b/0ylhSY/vLT1) <https://play.google.com/store/books/details?id=W8-5BQAAQBAJ>

24. [Santos M, Monteiro AL, Santos B. EQ-5D Brazilian population norms. Health Qual Life Outcomes. 2021;19: 162. doi:](http://paperpile.com/b/0ylhSY/lSuO)[10.1186/s12955-021-01671-6](http://dx.doi.org/10.1186/s12955-021-01671-6)

25. [Bailey HH, Janssen MF, Varela RO, Moreno JA. EQ-5D-5L Population Norms and Health Inequality in Colombia. Value Health Reg Issues. 2021;26: 24–32. doi:](http://paperpile.com/b/0ylhSY/56wJ)[10.1016/j.vhri.2020.12.002](http://dx.doi.org/10.1016/j.vhri.2020.12.002)

26. [Bailey H, Janssen MF, La Foucade A, Boodraj G, Wharton M, Castillo P. EQ-5D self-reported health in Barbados and Jamaica with EQ-5D-5L population norms for the English-speaking Caribbean. Health Qual Life Outcomes. 2021;19: 97. doi:](http://paperpile.com/b/0ylhSY/Xzt1)[10.1186/s12955-021-01734-8](http://dx.doi.org/10.1186/s12955-021-01734-8)

27. [Bailey H, Janssen MF, La Foucade A, Kind P. EQ-5D-5L population norms and health inequalities for Trinidad and Tobago. PLoS One. 2019;14: e0214283. doi:](http://paperpile.com/b/0ylhSY/FK02)[10.1371/journal.pone.0214283](http://dx.doi.org/10.1371/journal.pone.0214283)

28. [Hagens A, İnkaya AÇ, Yildirak K, Sancar M, van der Schans J, Acar Sancar A, et al. COVID-19 Vaccination Scenarios: A Cost-Effectiveness Analysis for Turkey. Vaccines (Basel). 2021;9. doi:](http://paperpile.com/b/0ylhSY/2tXpH)[10.3390/vaccines9040399](http://dx.doi.org/10.3390/vaccines9040399)

29. [Sandmann FG, Davies NG, Vassall A, Edmunds WJ, Jit M, Centre for the Mathematical Modelling of Infectious Diseases COVID-19 working group. The potential health and economic value of SARS-CoV-2 vaccination alongside physical distancing in the UK: a transmission model-based future scenario analysis and economic evaluation. Lancet Infect Dis. 2021;21: 962–974. doi:](http://paperpile.com/b/0ylhSY/ZQK4A)[10.1016/S1473-3099(21)00079-7](http://dx.doi.org/10.1016/S1473-3099(21)00079-7)

30. [Padula WV, Malaviya S, Reid NM, Cohen BG, Chingcuanco F, Ballreich J, et al. Economic value of vaccines to address the COVID-19 pandemic: a U.S. cost-effectiveness and budget impact analysis. J Med Econ. 2021;24: 1060–1069. doi:](http://paperpile.com/b/0ylhSY/Nyll4)[10.1080/13696998.2021.1965732](http://dx.doi.org/10.1080/13696998.2021.1965732)

31. [Kohli M, Maschio M, Becker D, Weinstein MC. The potential public health and economic value of a hypothetical COVID-19 vaccine in the United States: Use of cost-effectiveness modeling to inform vaccination prioritization. Vaccine. 2021;39: 1157–1164. doi:](http://paperpile.com/b/0ylhSY/raY0e)[10.1016/j.vaccine.2020.12.078](http://dx.doi.org/10.1016/j.vaccine.2020.12.078)
